# AV2 protein of tomato leaf curl Palampur virus promotes systemic necrosis in *Nicotiana benthamiana* and interacts with host Catalase2

**DOI:** 10.1038/s41598-018-19292-3

**Published:** 2018-01-19

**Authors:** Poonam Roshan, Aditya Kulshreshtha, Surender Kumar, Rituraj Purohit, Vipin Hallan

**Affiliations:** 1 Academy of Scientific & Innovative Research (AcSIR), CSIR-Institute of Himalayan Bioresource Technology (CSIR-IHBT) Campus, Palampur, HP 176061 India; 2 Plant Virology Lab, CSIR-IHBT, Palampur, HP 176061 India; 3 Biotechnology division, CSIR-IHBT, Palampur, HP 176061 India

## Abstract

Tomato leaf curl Palampur virus (ToLCPalV) is a whitefly-transmitted, bipartite begomovirus. Here, we demonstrated that ectopic expression of AV2 from a *Potato virus* X (PVX)-based vector accelerated systemic necrosis and reactive oxygen species (ROS) accumulation in *Nicotiana benthamiana*. Furthermore, 10 amino acids from N-terminal region of AV2 were found to be associated with the systemic necrosis symptom/phenotype. Mutational studies of ToLCPalV infectious clones lacking the AV2 revealed that AV2 is essential for the systemic movement of DNA-A, symptom severity and viral DNA accumulation. In a yeast two-hybrid assay, Catalase2 (Cat2) was found to associate with AV2 protein. Further, silencing of *Cat2* resulted in appearance of necrotic lesions on *N. benthamiana* and these plants were highly susceptible to ToLCPalV infection in comparison to control plants. Infection ToLCPalV on *Solanum lycopersicum* resulted in downregulation of *Cat2* transcripts, followed by accumulation of ROS and stress marker transcripts. The AV2 protein also suppressed *virus-induced gene silencing* (VIGS) of the *Phytoene desaturase* (*PDS*) gene. Our results show that AV2 is essential for the pathogenicity, systemic movement and suppression of gene silencing in the host. Altogether, our findings suggest that interactions between AV2 and Cat2 might play a crucial role in the establishment of ToLCPalV infection.

## Introduction

Viruses are biotrophic pathogens that utilize the cellular machinery of a host for successful invasion. Disease establishment relies on compatible or incompatible interactions between the virus and host factors. An incompatible interaction of a virulent *avr* protein (effector) with the R protein (plant receptor) can lead to the development of disease resistance against the invading pathogen^1^, triggering biochemical and physiological changes that are accompanied by the hypersensitive response and/or systemic acquired resistance. These cellular events lead to regulation/activation of defence-related plant genes, which results in cessation of viral replication at sites of entry or virus movement^1–3^. In contrast, a compatible interaction between a virus and host renders the plant susceptible to pathogen attack. The generation of reactive oxygen species (ROS), e.g., hydrogen peroxide (H_2_O_2_) and superoxide radical (O^2−^), also known as the oxidative burst, is considered to be the earliest response shown by plant cells to biotic stress^4^. To protect cells from the deleterious effects of H_2_O_2_, antioxidant enzymes such as catalase help to detoxify H_2_O_2_. Catalases are mainly present in peroxisomes and can be classified into three classes: Class I, which are abundant in photosynthetic tissues; Class II, which are present in vascular tissues; and Class III, which are expressed in reproductive tissues. Catalase1 (Cat1), Catalase2 (Cat2) and Catalase3 (Cat3) are type members of Classes III, I and II, respectively^5^. To counter viral attack, plants primarily deploy RNA-silencing mechanisms involving sequence-specific degradation of invading viral nucleic acids. The virus-derived dsRNAs are recognized, cleaved, and loaded onto the RNA-induced silencing complex (RISC); target the cognate viral RNAs for degradation^6^. Viruses encode suppressor(s)/pathogenicity protein(s) to suppress the antiviral defence response of the host^7–10^.

The genus *Begomovirus* of family *Geminiviridae* is one of the largest genera comprising of >325 members^11^. Members of the genus are ssDNA viruses; the genetic material is encapsulated in twinned icosahedral particles, may be organized monopartite or bipartite material (on the basis of genomic DNA circles) and replicates via rolling circle replication (RCA)^12^. Tomato leaf curl Palampur virus (ToLCPalV) is a bipartite begomovirus that infects tomato, cucurbits and beans in Asia^13–15^. DNA-A possesses six open reading frames (ORFs): AV1 and AV2 are present on the virion sense strand, and AC1, AC2, AC3 and AC4 are present on the virion antisense strand. The DNA-B component comprises the BV1 and BC1 ORFs on the virion sense and antisense strands, respectively. These ORFs encode the coat protein (AV1), precoat protein (AV2), replication initiator protein (AC1), transcription activator protein (AC2), replication enhancer protein (AC3), AC4 protein, nuclear shuttle protein (BV1) and movement protein (BC1). A ~14-kDa protein encoded by the AV2 ORF of DNA-A overlaps with the AV1 ORF at its C-terminus^13^. Regarding monopartite tomato-infecting begomoviruses, the homologous V2 ORF/movement protein of tomato yellow leaf curl virus (TYLCV), tomato yellow leaf curl China virus (TYLCChV), tomato yellow leaf curl Java virus (TYLCJV) and tomato yellow leaf curl Sardinia virus (TYLCSV) have been reported to suppress RNA silencing by the host^10,16–19^. Specifically, TYLCV V2 has been shown to bind to SGS3 or dsRNA to prevent amplification of siRNA during RNA silencing^9,20^. The V2 protein of TYLCV targets a cysteine protease (a papain-like cysteine protease) involved in plant defence^21^ and also mediates the nucleo-cytoplasmic shuttling of viral DNA in host^22^. Although viral suppressors have evolved to counter the RNA silencing mechanism, it is clear that they can also target other defence components of the host.

In the present study, we examined the role of the ToLCPalV AV2 protein with regard to the pathogenesis of infection. Here, we show that AV2 interacts with the free radical-scavenging Cat2 protein and induces systemic necrosis-like responses by regulating the levels of host *Cat*2 transcript. AV2 was also found to suppress virus-induced gene silencing by the host.

## Results

### The N-terminal region of the AV2 protein is involved in systemic necrosis and ROS generation in *Nicotiana benthamiana*

**Figure 1:**
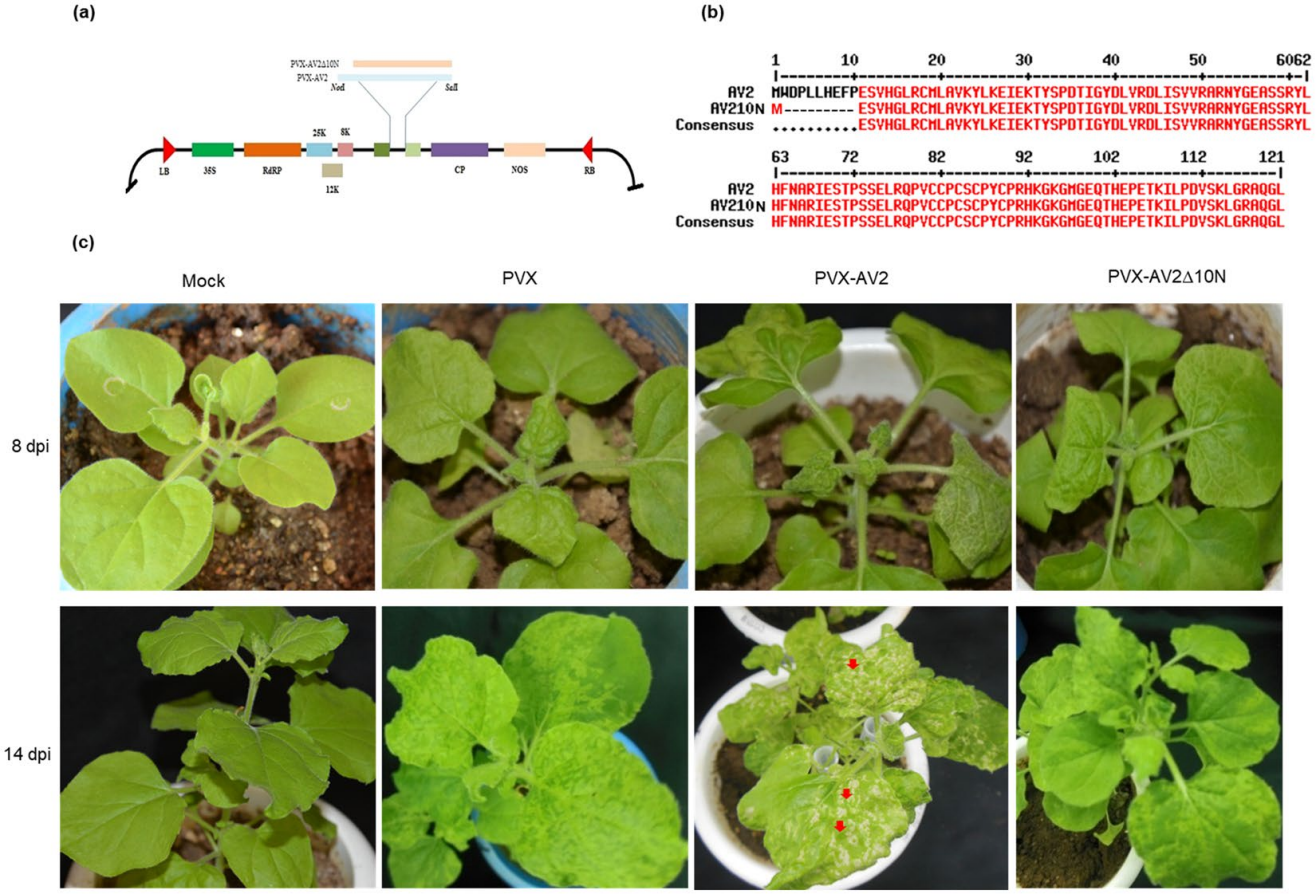
(**a**) Diagrammatic representation of *AV2* full-length and N-terminal mutant gene fragments in the pGR106 vector. (**b**) Amino acid alignment of the full-length (AV2) and N-terminal-mutated (AV2Δ10N) proteins, depicting the deletion of 10 amino acids. (**c**) Symptomatic plants inoculated with PVX, PVX-AV2 and PVX-AV2Δ10N at 8 and 14 dpi, respectively, showing the necrotic lesions in PVX-AV2 infiltrated plants.

**Figure 2:**
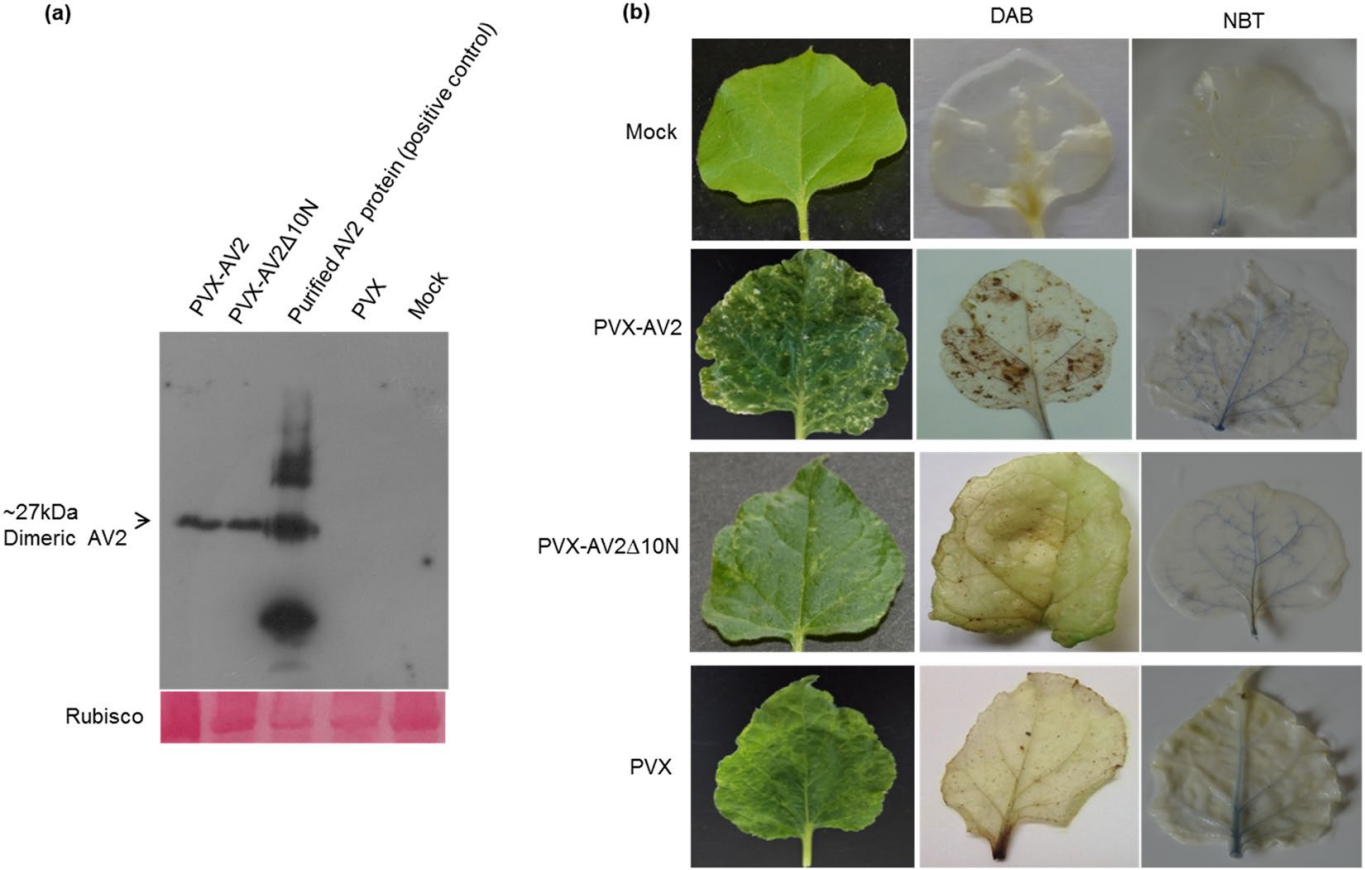
(**a**) Western blot was performed with AV2 antisera to detect the expression of AV2 in *N. benthamiana* leaves samples infiltrated with PVX-AV2, PVX-AV2Δ10N at 14 dpi. PVX, mock infiltrated samples; and purified AV2 protein were used as negative control and positive controls, respectively. (**b**) DAB and NBT staining for detection of H_2_O_2_ and O_2_^−^ radicals in PVX-AV2, PVX-AV2Δ10N and PVX inoculated *N*. *benthamiana* at 14 dpi, showing the higher accumulation of ROS in PVX-AV2 infiltrated leaves.

Primers were designed to generate an N-terminal deletion mutant lacking the 10 amino acids from N-terminal of AV2 (AV2Δ10N). Both the full-length and the deletion mutant were cloned into a PVX-based vector (pGR106) under control of the CaMV35S promoter. PVX-AV2 (366 bp), N-terminal deletion mutant PVX-AV2Δ10N (336 bp), (Fig. 1b) and the empty vector (pGR106) were inoculated into four-week-old *N. benthamiana* plants through agrobacterium-mediated infiltration (GV3101 strain). At 8 days post-inoculation (dpi), typical mild mosaic symptoms were observed in plants infiltrated with the pGR106 vector (control), PVX-AV2 and PVX-AV2Δ10N. By 14 dpi, the PVX-AV2-infiltrated plants displayed severe mosaic and leaf deformations with necrotic lesions. In contrast, the PVX- and PVX-AV2Δ10N-infiltrated plants exhibited mosaic-like symptoms with dark green patches (Fig. 1c). As anticipated, the AV2 protein was detected in both the PVX-AV2- and PVX-AV2∆10N-infiltrated plants in western blot analysis, with corresponding bands of ~28 kDa (dimer), (Fig. 2a). As the appearance of necrotic lesions is usually associated with overproduction of ROS, such as H_2_O_2_; *N. benthamiana* leaves (14 dpi) were treated with a 3,3′-diaminobenzidine (DAB)-HCl solution. PVX-AV2-infiltrated leaves displayed accumulation of a brown-coloured product, whereas the PVX-AV2∆10N- and PVX-infiltrated plants did not develop this brown colour after DAB-HCl treatment. Subsequently, after nitroblue tetrazolium (NBT) staining for the detection of superoxide radical (O_2_^−^), accumulation of a blue-coloured product was observed in *N. benthamiana* leaves expressing PVX-AV2 (Fig. 2b). These results indicate that ectopic expression of AV2 resulted in overproduction of ROS. We also overexpressed the AV2 protein under the control of the CaMV35S promoter in *N. benthamiana* using leaf disc transformation. We selected 15 regenerated transgenic plants overexpressing the AV2 protein after hygromycin selection; T0 plants were grown under greenhouse conditions, and the presence of a transgene was confirmed by PCR amplification using gene-specific primers. In the initial stages of hardening, the T0 lines displayed a leaf-curling phenotype; however, none of the plants were able to survive at the later stages (Supplementary Fig. 1). These results suggest that stable overexpression of the AV2 protein is detrimental to the normal growth of this plant.

### The AV2 protein is indispensable for causing typical virus symptoms during ToLCPalV infection

**Figure 3:**
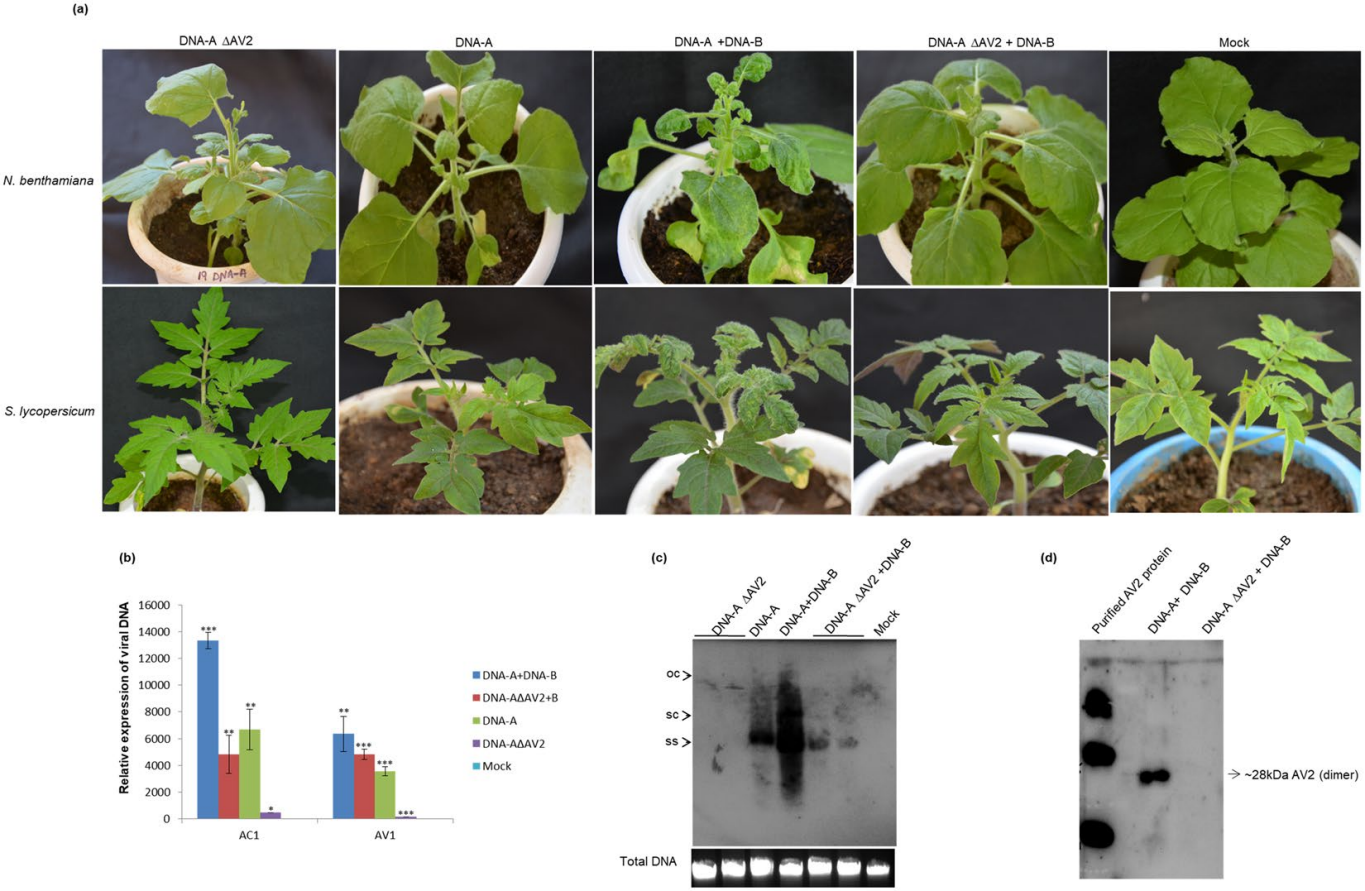
(**a**) Infectivity analysis of wild-type and mutant ToLCPalV on *N. benthamiana* and *S. lycopersicum*. Plants were inoculated with agro-infectious clones of respective constructs and photographed at 14 dpi. Plants infiltrated with DNA-A∆AV2; DNA-A alone and DNA-A∆AV2+DNA-B were asymptomatic, whereas DNA-A+DNA-B-infiltrated plants developed severe downward leaf-curling symptoms. (**b**) Real-time expression for relative quantification of viral DNA in inoculated leaves at 3 dpi. Asterisk on the top of the bars indicate statistical significant difference using Student’s-T test between mock and infected samples (*(P < 0.05), **(P < 0.01) and ***(P < 0.001). *EF*1α was used as internal control and experiment was repeated twice with three biological and technical replicates. (**c**) Southern blot for viral DNA accumulation with a *CP* gene-specific probe. DNA was isolated from newly emerged leaves of *N. benthamiana* at 21 dpi; higher accumulation of viral single-stranded (ss DNA), super coiled (sc DNA) and open circular (ocDNA) forms was observed in DNA-A+DNA-B-infiltrated leaves. (**d**) Western blot with AV2 antisera for detection of the AV2 expression in DNA-A+DNA-B- and DNA-A∆AV2+DNA-B-infiltrated *N. benthamiana*; purified AV2 protein was used as the positive control.

The agrobacterium-mediated inoculation of infectious clones of ToLCPalV (DNA-A+DNA-B) in *N. benthamiana* and *S. lycopersicum* resulted in downward leaf curling, vein thickening, reduced leaf lamina and internode shortening^23^. However, infectious clones of ToLCPalV harbouring a mutated AV2 gene (DNA-A∆AV2+DNA-B) failed to cause such symptoms (Fig. 3a). Total DNA was extracted from infiltrated plants, and RCA was performed; RCA products were then sequenced with DNA-A-specific primers to confirm the integrity of the mutation in viral progeny. The sequencing results revealed that mutation of the AV2 gene did not alter the remaining DNA-A sequence. The infiltrated leaves were analyzed at 3 dpi to assess the virus replication by real time PCR. The results revealed higher accumulation of AC1 and AV1 transcripts in DNA-A+DNA-B inoculated leaves in comparison to those infiltrated with DNA-A∆AV2+DNA-B (Fig. 3b). These results indicated the role of AV2 in replication of virus. Southern hybridization was also performed to assess the systemic movement of the virus at 21 dpi. The results showed higher accumulation of single-stranded (ss), super coiled (sc) and open circular (oc) DNA forms, indicating that virus was able to efficiently replicate and move systemically in plants infiltrated with DNA-A+DNA-B. Moreover, among all DNA forms, the amount of ssDNA was highest in these plants at 21 dpi in comparison to plants infiltrated with DNA-A alone or DNA-A∆AV2+DNA-B. In contrast, viral DNA was not detected in plants infiltrated with DNA-A∆AV2 alone, suggesting that AV2 affects the replication as well as the viral DNA accumulation (Fig. 3c). Further, dimeric form of AV2 was detected in the ToLCPalV infected leaves in an immunoblot analysis (Fig. 3d).

We also analysed the presence of H_2_O_2_ and O_2_^-^ radical in virus-infected *S. lycopersicum* leaves and found that plants inoculated with DNA-A∆AV2+DNA-B showed reduced ROS accumulation compared to wild type virus inoculated plants at 21 dpi (Supplementary Fig. 2a). In addition, ToLCPalV-infected *N. benthamiana* leaves showed a 2-fold decrease in total Catalase activity in comparison to mock-infiltrated plants (Supplementary Fig. 2b).

### AV2 acts as a transcriptional activator in yeast and interacts with host Catalase2

**Figure 4:**
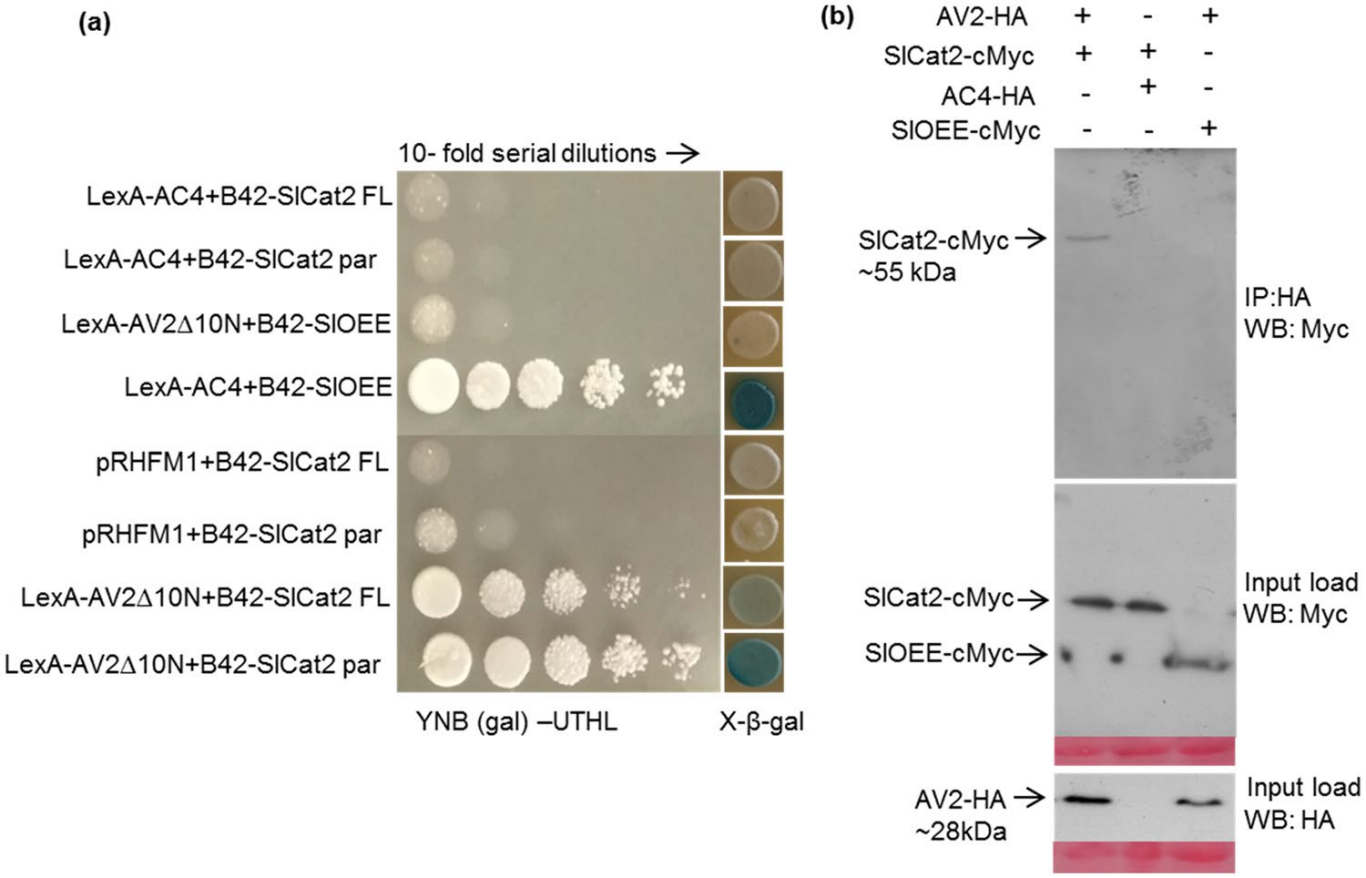
Interaction between AV2 and Cat2 in a Yeast two-hybrid system. The yeast strain EGY48 harbouring the indicated plasmids were spotted with 10-fold serial dilutions on YNB (gal) –ura –trp –his –leu medium and β-galactosidase assay was performed on YNB (gal) –ura –trp –his medium supplemented with β-X-gal. Abbreviations: B42-SlCat2 FL (full length Cat2 in pJG4-5 vector), B42-SlCat2 par (partial Cat2 in pJG4-5 vector), B42-SlOEE (OEE in pJG4-5 vector) and LexA-AV2∆10N (AV2 lacking 10 amino acids in pEG202 vector). (**b**) Co-immunoprecipitation of the complex (AV2-HA+Myc-SlCat2) was carried out using an anti-HA antibody (raised in rabbit) and the immunoprecipitated protein (Myc-SlCat2) was detected using an anti-cMyc antibody (raised in mouse). The Myc-tagged SlOEE and HA-tagged AC4 constructs were used as a negative control. Abbreviations: AV2-HA (AV2 in pSPYCE(M) vector), SlCat2-cMyc (Cat2 in pSPYNE(R)173 vector), AC4-HA (AV2 in pSPYCE(M) vector) and SlOEE-cMyc (OEE in pSPYNE(R)173 vector.

To identify host factors interacting with the AV2 protein, yeast two-hybrid (Y2H) analysis was performed using a tomato cDNA library cloned in the pJG4-5 vector (B42); (a kind gift from Prof. Yedidya Gafni, Israel). Initial assessment of the bait protein’s auto-activation potential revealed that the AV2 protein possesses transactivation activity. Therefore, an N-terminal deletion mutant of AV2 cloned in the pEG202 vector (LexA-AV2Δ10N) lacking this activity was used for library screening. We obtained 4 × 10^6^ yeast transformants, and six potential candidate clones were selected using yeast nitrogen base galactose lacking uracil, tryptophan, histidine and leucine (YNB (gal) -ura -trp -his –leu) medium. All clones were sequenced, and one of the clones showed similarity to the *Catalase2* sequence of *S. lycopersicum* (Accession number: AF112368) in a BLAST search. Cat2 is a tetrameric enzyme, in which each monomer consists of a haeme-containing prosthetic group, catalyses conversion of H_2_O_2_ into water and molecular oxygen. Although the majority of Cat isoforms localize to peroxisomes, the enzyme has recently been found in the cytoplasm, chloroplasts and mitochondria^24–26^. As the *Cat2* isolated from the cDNA library was partial, we amplified a full-length *Cat2* from *S. lycopersicum* (*SlCat2*) by reverse transcription polymerase chain reaction (RT-PCR) method and cloned it into the pJG4-5 (prey) vector. Interaction between LexA-AV2Δ10N and full-length SlCat2 (B42-SlCat2 FL) was also confirmed in yeast. The absence of non-specific interaction of Cat2 (full length and partial) was confirmed using bicoid homeodomain (pRHFM1), which was unable to grow on leucine deficient medium. The LexA-AC4 and B42-SlOEE plasmids were used as bait and prey negative controls, respectively. The interaction of LexA-AC4 and B42-SlOEE was used as a positive control (unpublished data) in this study. Y2H interactions were confirmed on both leucine deficient selective medium and β-X-gal selective medium (Fig. 4a). The complete nucleotide sequence of the *SlCat2* gene (1479 bp) was submitted to the European Molecular Biology Laboratory (EMBL) database (accession number LN880542).

A docking 3D model of SlCat2 and AV2 was developed using the Hex 8.0.0 server, and a schematic representation of the receptor (SlCat2) and ligand (AV2) has been depicted (Supplementary Fig. 3). AV2 interacts with SlCat2 via residues Asn13, Asn28, Glu57 and Gln253 through hydrophobic interactions and Tyr4, Ser10, Asn27, Asn29, Glu57, Arg58, Tyr256, Asp257, Phe315, Asn247, Ser249, and Gly111 through hydrogen bonding. These interacting amino acids of SlCat2 correspond to the haeme-binding pocket and tetramer interface involved in the conversion of H_2_O_2_ into water and O_2_.

### *In planta* interaction of AV2 with SlCat2

**Figure 5:**
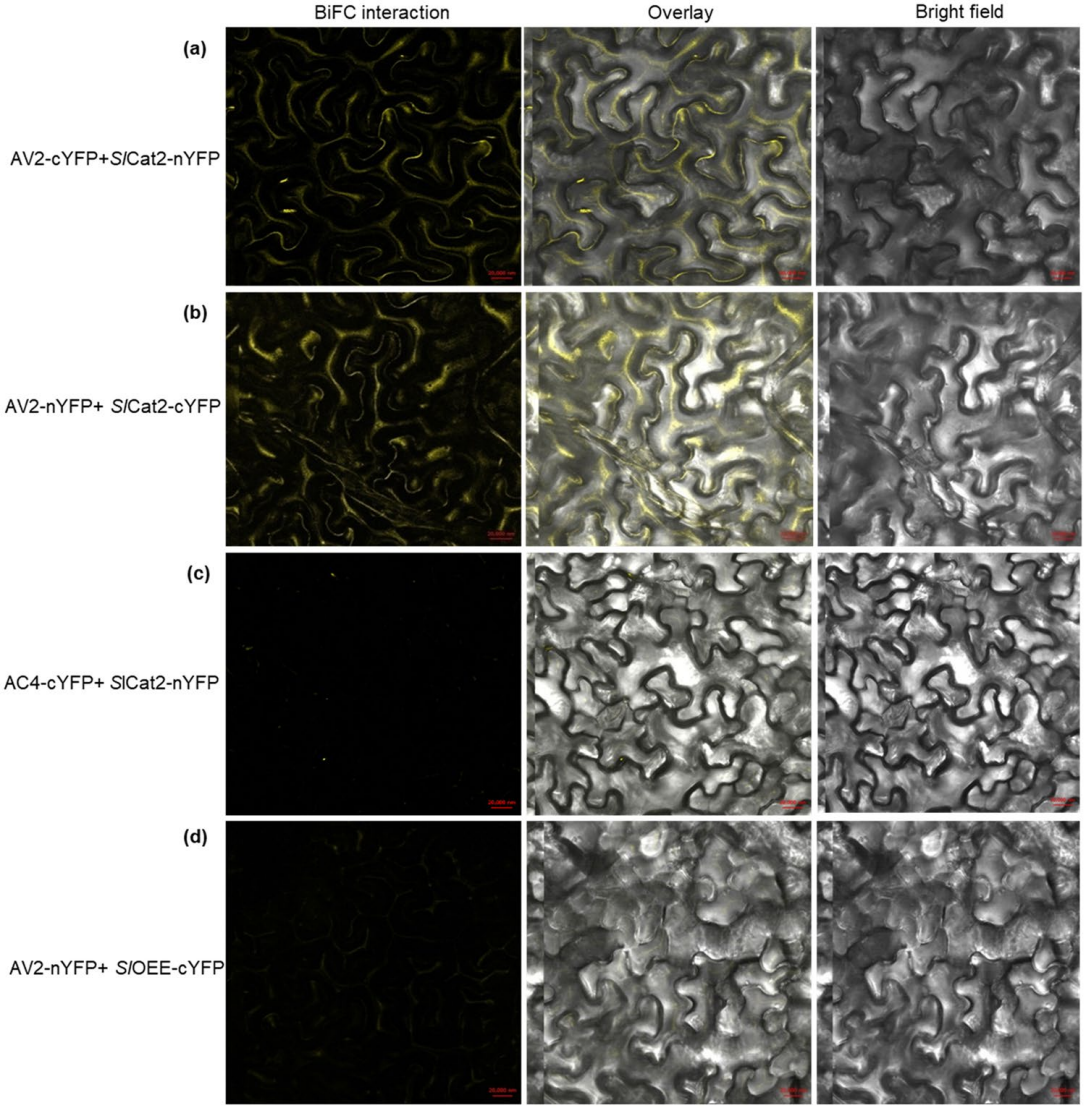
BiFC image of *N. benthamiana* leaves co-expressing (**a**) AV2-cYFP+SlCat2-nYFP; (**b**) AV2-nYFP+SlCat2-cYFP; (**c**) AC4-cYFP+SlCat2-nYFP (negative control) and; (**d**) AV2-nYFP+SlOEE-cYFP (negative control). AV2-SlCat2 interaction resulted in reconstitution of YFP fluorescence in the cytoplasm and along the cell periphery. Images were captured on a 20 × magnification scale. Abbreviations: AV2-cYFP (AV2 in pSPYCE(M) vector), AV2-nYFP (AV2 in pSPYCE(MR) vector), SlCat2-nYFP (Cat2 in pSPYNE(R)173 vector), SlCat2-cYFP (Cat2 in pSPYNE173 vector), AC4-cYFP (AC4 in pSPYCE(M) vector) and SlOEE-cYFP (OEE in pSPYNE173 vector).

Leaf extracts of *N. benthamiana* transiently expressing SlCat2-cMyc, AV2-HA, SlOEE-cMyc, AC4-HA in bimolecular fluorescence complementation (BiFC) vectors were used to perform an *in vivo* pull-down assay. An anti-HA antibody was used to capture the protein complex, which was then bound to protein G magnetic beads. SlCat2 (~55 kDa) was detected by western blotting using an anti-Myc antibody (Fig. 4b). For further confirmation of the Y2H interaction, *in vivo* interaction between AV2 and SlCat2 was visualized using BiFC, which is based on reconstitution of yellow fluorescent protein (YFP) due to association between non-fluorescent halves. Gene segments encoding the N- and C-terminal fragments of YFP were fused to the *AV2* or *Cat2* gene and co-infiltrated on the underside of *N. benthamiana* leaves. Infiltration with AV2-cYFP+ SlCat2-nYFP and AV2-nYFP+SlCat2-cYFP constructs resulted in strong YFP signals in the cytoplasm and along the cell margins. Conversely, no fluorescence was detected in leaves infiltrated with SlCat2-nYFP+AC4-cYFP and AV2-nYFP and SlOEE-cYFP, respectively (Fig. 5). These results confirm that AV2 interacts with host Cat2 in the cytoplasm.

### AV2 exists as a dimer *in vivo*

Lex-A-based Y2H studies revealed that AV2 possesses transactivation activity. Therefore, the split-ubiquitin-based Y2H system was used to study self-interaction of AV2. Split ubiquitin-based Y2H is suitable for bait proteins that show auto-activation/transactivation activity. To assess auto-activation potential, pDHB1-AV2 (bait) was transformed along with pPR3N (the prey vector) into the strain NMY51. The transformed yeast colonies did not grow on yeast nitrogen base glucose lacking adenine, leucine, tryptophan and histidine (YNB (glu) -ade - leu -trp –his) medium supplemented with 10 mM 3-AT. In contrast, yeast transformed with the pDHB1-AV2 (bait) and pPR3N-AV2 (prey) plasmids were able to grow on YNB (glu) -ade -leu -trp -his medium supplemented with 10 mM 3-AT, indicating that AV2 interacts with itself in yeast (Supplementary Fig. 4).

### AV2 is a suppressor of post-transcriptional gene silencing (PTGS)

The abovementioned activity of AV2 (pathogenicity and symptom determinant) prompted us to evaluate the RNA-silencing suppressor activity of this protein. The agrobacterium strain GV3101 transformed with TRV2-AV2, TRV2-Phytoene desaturase (PDS; VIGS control), TRV2-P19 (positive control) or TRV1 (control) was used in this study. At 14 dpi, plants co-infiltrated with TRV2-PDS+TRV1 displayed a bleaching phenotype in systemic leaves due to the production of siRNAs against the PDS gene, which resulted in silencing of this gene. In the case of plants co-inoculated with TRV2-AV2+TRV2-PDS+TRV1, reduced/suppressed bleaching was observed, indicating interference of AV2 by RNA silencing machinery. *N. benthamiana* inoculated with TRV2-PDS+TRV2-P19+TRV1 (the suppressor positive control) did not exhibit a bleaching phenotype (Supplementary Fig. 5a). Suppression of *PDS* silencing was further confirmed by semi-quantitative PCR analysis (Supplementary Fig. 5b). Western blot analysis of total protein from TRV2-AV2+TRV2-PDS+TRV1, TRV2-PDS+TRV2-P19+TRV1, TRV2-PDS+TRV1 and mock-infiltrated plants was performed to detect the presence of AV2 (Supplementary Fig. 5c), and the results revealed the presence of the dimeric form of the viral protein.

### Expression of AV2 leads to accumulation of defence-related transcripts involved in priming of the salicylic acid pathway

**Figure 6:**
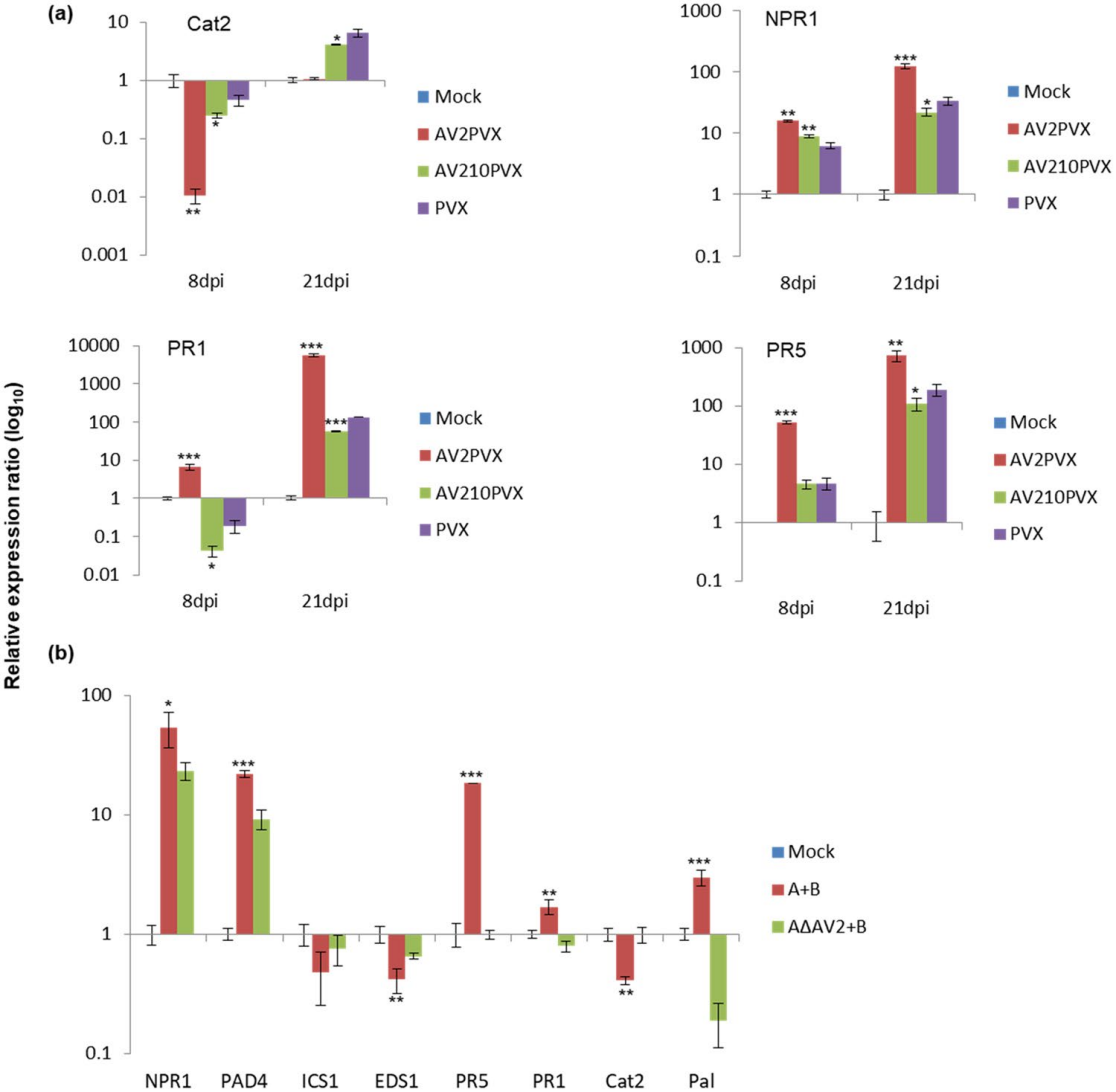
(**a**) Relative expression of defense-related transcripts (*Cat2*, *NPR1*, *PR1* and *PR5*) in systemic leaves of *N. benthamiana* inoculated with PVX-AV2, PVX-AV2Δ10N and PVX at 8 and 21 dpi. (**b**) Relative expression of SA-responsive genes in systemic leaves of *S. lycopersicum* infected with ToLCPalV (A+B) and ToLCPalV∆AV2 (A∆AV2+B) at 21 dpi. The EF1α was used to normalize the transcripts. Asterisk on the top of the bars depict the statistical significant differences between mock and infected samples using Student’s-T test(*(P < 0.05), **(P < 0.01) and ***(P < 0.001). All experiments were performed with three biological and technical replicates.

As ectopic expression of AV2 resulted in formation of necrotic spots and accumulation of ROS, we analysed the expression levels of various genes related to defence responses, including *NPR1*, *PR1*, *PR5*, *and Cat2*, by quantitative RT-PCR (qRT-PCR). Total RNA was isolated from *N. benthamiana* leaves infiltrated with the PVX-AV2, PVX-AV2Δ10N and pGR106 vectors at 8 and 21 dpi. In leaves expressing PVX-AV2, expression of *PR1*, *PR5* and *NPR1* was 6-, 52- and 15-fold upregulated, respectively, at 8 dpi, whereas *Cat2* gene expression was found to be 100-fold downregulated (Fig. 6a). At 21 dpi, no significant changes in *Cat2* transcript levels were observed in PVX-AV2-inoculated plants, though levels were increased up to 4- and 6-fold in PVX-AV2Δ10N- and PVX vector-inoculated plants, respectively. Interestingly, the expression levels of *PR1*, *PR5* and *NPR1* were highest in PVX-AV2-infiltrated leaves (Fig. 6a). These results indicated that an increase in ROS is accompanied by upregulation of defence-related transcripts (*PR1*, *PR5* and *NPR1*) followed by development of the systemic necrosis phenotype in *N. benthamiana* expressing the AV2 protein.

Total RNA was also isolated from DNA-A+DNA-B−, DNA-A∆AV2+DNA-B− and mock-inoculated *S. lycopersicum* plants at 21 dpi, and expression analysis of various genes involved in salicylic acid (SA)-based defence signalling was performed. Expression of the SA precursor isochorismate synthase (ICS1) was downregulated by 2-fold, whereas 2.9-fold upregulation in phenylalanine ammonia lyase (*PAL*) transcript levels was observed. Expression analysis of two important genes that act upstream of the SA pathway revealed upregulation of *PAD4* (*Phytoalexin-deficient protein 4*) by 21-fold and downregulation of *EDS1* (*Enhanced disease susceptibility 1*) by 2.4-fold. However, in DNA-A+DNA-B infiltrated plants, the genes *NPR1*, *PR1* and *PR5* were upregulated by 53-, 1.6- and 18-fold, respectively, and *Cat2* was downregulated by 2.6-fold (Fig. 6b).

### Silencing of *Cat2* promotes ToLCPalV infection and leads to development of necrotic lesions in *N. benthamiana*

**Figure 7:**
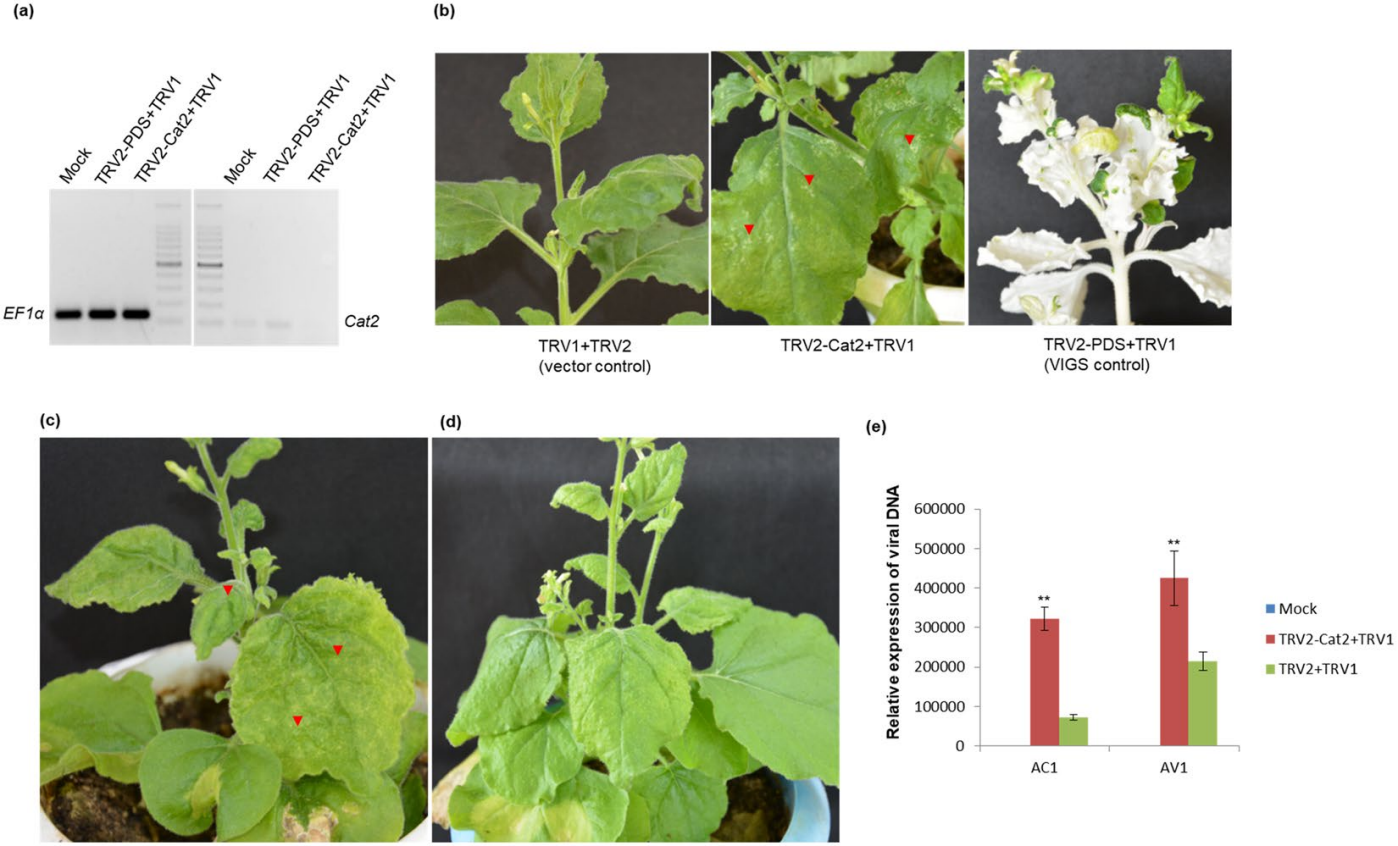
(**a**) Semi-quantitative PCR for the confirmation of *Cat2* silencing in *N. benthamiana*; *EF*1α was used to normalise the cDNA samples (**b**) Silencing of *Cat2* resulted in the appearance of necrotic lesions in comparison to the vector control. (**c**) *Cat*2 silenced plants showed exaggerated symptoms (downward leaf curling) along with necrotic lesions; (**d**) in comparison to vector control upon ToLCPalV challenge. (**e**) Real-time expression analysis for the relative quantification of viral DNA in *Cat*2 silenced plants after ToLCPalV challenge. Asterisk on the top of the bars indicate statistical significant differences using Student’s-T test between mock and infected samples (*(P < 0.05), **(P < 0.01) and ***(P < 0.001). The experiment was repeated with three biological and technical replicates.

Transient silencing of *Cat2* was achieved using the *Tobacco rattle virus vector* (TRV), VIGS vector. At 10 dpi, *N. benthamiana* infiltrated with TRV2-PDS+TRV1 displayed a bleaching phenotype, confirming VIGS. Silencing of *Cat2* was also confirmed by semi-quantitative PCR (Fig. 7a), and *Cat2*-silenced plants displayed cell death-like symptoms (Fig. 7b). At 10 dpi of the VIGS experiment, *Cat2*-silenced (TRV2-Cat2+TRV1) and vector control (TRV1+TRV2) plants were challenged with ToLCPalV, as a result the former (Fig. 7c) displayed severe symptoms in comparison to the latter (Fig. 7d). Furthermore, qRT-PCR based relative quantification of *AC1* and *AV1* ToLCPalV genes using equal quantities of total plant DNA and specific primers was performed to determine viral DNA load. It was observed that silencing of the *Cat2* gene resulted in higher accumulation of viral DNA compared with TRV-infiltrated plants (Fig. 7e). Based on these observations, it is clear that silencing of the *Cat2* gene enhanced the susceptibility of *N. benthamiana* to ToLCPalV infection and promoted cell death-like symptoms.

## Discussion

In plants, various defence mechanisms are critical for the defence against invading pathogens. Viruses that belong to the *Begomovirus* genus have been reported to cause dramatic economic losses^27^. As stated in the Introduction, the homologous V2 performs the role of a movement protein in monopartite begomoviruses. In bipartite begomoviruses, this function has been replaced by the BC1 protein of DNA-B, suggesting the origin of New World begomoviruses from Old World begomoviruses. The focus of the present study was therefore to decipher the role of the AV2 protein of ToLCPalV during disease establishment in *N. benthamiana* and *S. lycopersicum*. The PVX vector-based ectopic expression system has been developed to determine the pathogenicity of viral proteins^10,17,28,29^. In our study, we found that the ectopic expression of AV2 via the PVX vector resulted in the formation of necrotic lesions on the *N. benthamiana* leaves. In this context, it has been previously demonstrated that certain domains of viral proteins are essential for pathogenicity^28,30^. Similar necrosis-like symptoms were also observed in *N. benthamiana* overexpressing the TGB3 and polymerase domain of the RdRp protein of the pepino mosaic virus^31^. In the present study, we showed that 10 amino acids of the N-terminal region are essential for the development of necrotic symptoms, suggesting role in the symptom severity. Plants have developed several mechanisms to limit virus proliferation, and in such cases, the hypersensitive response (HR) or programmed cell death (PCD) are responsible for restricting spread of the virus. After viral invasion, plant cells undergo physiological changes, such as increased Ca^2+^ levels, ROS generation, hormone signalling pathway activation, and localized PCD^32^. The transient expression of the AV2 protein resulted in appearance of necrosis phenotype in the upper systemic leaves of *N. benthamiana*. The cell death or necrosis-like symptoms on upper systemic non-inoculated leaves at later stages of infection, accompanied by altered expression of defence-related genes, ROS production and PCD has been recognized as systemic necrosis^3^. Previous studies have shown that both systemic necrosis and HR exhibit similar biochemical and physiological changes at cellular level. Unlike HR, systemic necrosis is observed in the later stages of infection and do not hinder virus multiplication, thereby leading to susceptible infection^33,34^.Y2H screening and *in vivo* assays demonstrated that AV2 interacts with host SlCat2 (an enzyme present in photosynthetic tissue). Moreover, *Cat2* gene expression was reduced in *N. benthamiana* transiently overexpressing the AV2 protein, suggesting that downregulation of *Cat2* resulted in enhanced ROS levels accompanied by the appearance of necrotic lesions. These results also suggest that the AV2 protein might promote viral infection by reducing the levels of *Cat2* transcript as well as CAT activity in the host. Previous studies shown that CMV infection also results in downregulation of the *Cat3* gene, followed by systemic necrosis and enhanced virus accumulation in the host plant^35,36^. Conversely, TGBp1 of pepino mosaic virus has been shown to interact with host Cat1, promoting viral infection, accompanied by reduced H_2_O_2_ levels^37^.

Infectivity analysis of ToLCPalV and mutant ToLCPalV (ToLCPalV∆AV2) in host plants revealed that presence of the AV2 protein is essential for downward leaf curling, leaf epinasty and interveinal chlorosis. Moreover, virus progeny sequencing revealed that the reading frame of the CP ORF is unaffected by truncation of the AV2 ORF. Furthermore, absence of viral DNA in leaf samples infiltrated with DNA-A lacking the AV2 protein (DNA-A∆AV2) indicated a role for AV2 in the systemic movement of DNA-A. The absence of viral dsDNA form in plants infiltrated with DNA-A∆AV2+DNA-B enabled us to speculate that expression of AV2 affect the accumulation of viral DNA, thereby reducing the multiplication of virus. These findings also suggested that DNA-B might have partially complemented the movement functions of AV2 protein. It has been already known that replication of begomovirus DNA utilizes the dsDNA intermediates via RCR and the accumulation of both ssDNA and dsDNA (oc and sc) is crucial for the multiplication of the virus^38^. In previous studies, it has been shown that mutations in AV2/V2 ORF alone did not affect systemic spread of the virus and instead reduced viral DNA accumulation^39,40^. In contrast, a study revealed that mutations in *AV2* lead to symptomless infection and reduced viral DNA accumulation during TYLCV infection^41^. We found that plants infiltrated with DNA-A∆AV2+DNA-B remained asymptomatic, and these results indicate that the AV2 protein is a determinant of symptom severity. In support of our findings, the nuclear shuttle protein (NSP) of tomato leaf curl New Delhi Virus (ToLCNDV), the V2 protein of TYLCJV, the BV1 protein of bean dwarf mosaic virus (BDMV) and the βC1 protein of TYLCChV has been reported to be associated with disease development in plants^18,29,42,43^.

In general, plants respond to biotic stress by accumulating PR proteins, which has been shown to play a significant role in activation of defence pathways. For example, induction of *PR1* and *PR5* transcripts has been associated with production of SA against the biotrophic fungus^44,45^, and we found *PR1*, *PR5* and *NPR1* transcripts to be highly upregulated in plants transiently expressing the AV2 protein. Similar studies have been done which demonstrated that compatible interaction between plantago asiatica mosaic virus and its host involves PCD, leading to the systemic necrosis associated with induction of *PR* genes^34^. In another study, the expression of a stress marker (PR1) gene was induced during transient expression of a pathogenicity determinant protein (P25) from beet necrotic yellow vein virus^46^. Similarly, an interaction between a viral protein and necrosis-inducing factor (TuN1) of *Arabidopsis thaliana* caused necrosis in the plant, followed by expression of defence-related genes (PR1 and PR5)^47^. NPR1 exists as an oligomer in the cytosol. However, upon pathogen infection, it is reduced to a monomer and then transported into the nucleus to activate defence-related genes^48,49^. As NPR1 is a positive regulator of SA signalling, we analysed the expression levels of genes encoding SA precursors in ToLCPalV-infected leaves. We found that *PAL1* expression was upregulated during the ToLCPalV infection in *S*. *lycopersicum*, whereas expression of ICS1 was downregulated. ICS1 is known to contribute for the majority of pathogen induced SA accumulation in the infected host in comparison to PAL pathway^50^. EDS1 and PAD4 act upstream of the SA signalling pathway and act as positive feedback loop for SA induction. EDS1 also participates in the generation of the SAR signal both in proximal and distal tissues^51,52^. However, we found that the *EDS1* gene was downregulated and the *PAD4* and *NPR1* genes upregulated, indicating differential regulation of *EDS1*, *NPR1* and *PAD4* gene expression during ToLCPalV infection. Our results indicated that the downregulation of EDS1 might be compromising the induction of SA in virus infected host. A previous study also reported that CMV infection induces SA accumulation but that 2b protein of the virus overcomes SA-induced resistance^53^. Our previous study has demonstrated the localization of AV2 protein in both nucleus and cytoplasm of the host cell^54^. Based on these observations, we suggest that AV2 might serve as an elicitor for activating the SA pathway by altering the expression of defense related genes in nucleus as well as in cytoplasm of host. The development of typical virus symptoms in the presence of AV2 indicates that it also could be one of the viral proteins responsible for symptom severity.

In the present study, the AV2 protein was able to suppress silencing of the *PDS* gene, and western blot analysis of infiltrated plants revealed that AV2 was present in the dimeric form. AV2 also showed transactivation activity and interaction with its own in yeast. Although AV2 can exist as monomers, homodimers and homo-oligomers *in vitro*, the presence of AV2 protein dimers *in vivo* suggest that suppressor activity is also associated with the dimeric form. Previous studies have also shown that suppressors of gene silencing, such as the 2b protein of cucumber mosaic virus (CMV) and the AC2 protein of mungbean yellow mosaic virus (MYMV), also possess transactivation activity^8,55^. It has long been known that viruses employ pathogenicity determinant/factors for disease induction in susceptible hosts by interacting with host proteins. For instance, the βC1 protein of begomovirus betasatellite acts as an RNA suppressor and pathogenicity determinant; overexpression of βC1 leads to developmental aberrations in the host^56–58^. The results of ToLCPalV challenge in *Cat2*-silenced benthamiana leaves revealed their enhanced susceptibility, which indicates that *Cat2* could be an important host factor targeted by the virus for successful infection. Interestingly, *Cat2*-silenced *N. benthamiana* plants developed necrotic lesions similar to leaves expressing AV2 protein. Similar results were obtained in *Cat1*-deficient tobacco during pathogen stress^59,60^.

**Figure 8:**
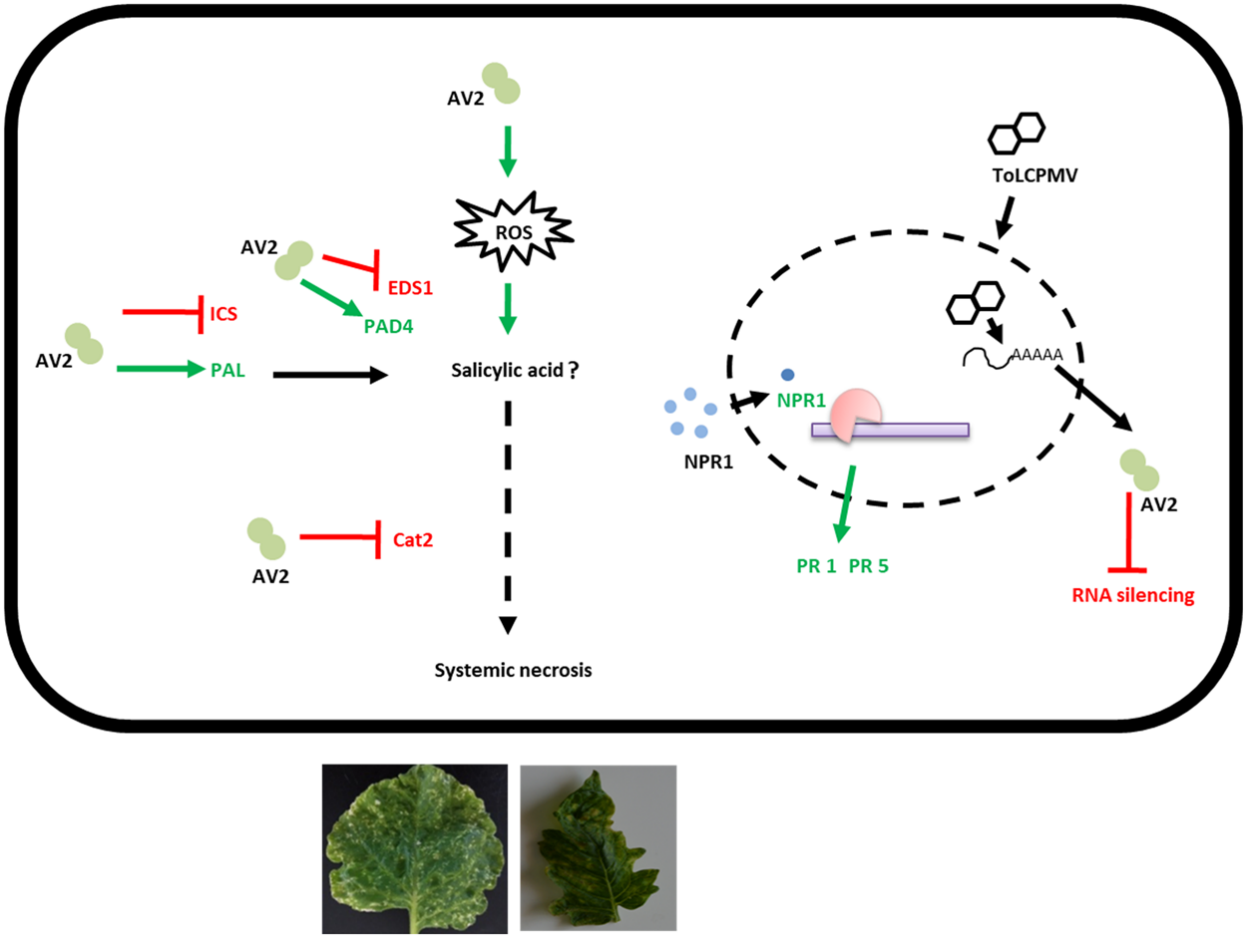
A schematic hypothesis for the role of the AV2 protein during ToLCPalV infection. ROS are generated as a result of virus entry into the cell. Expression of PAD4 might prime salicylic acid (SA) via the PAL pathway. SA priming might lead to expression of NPR1, which eventually induces expression of stress markers (PR1 & PR5). The downregulation of EDS1 might have weakened the SA accumulation and renders the host susceptible for the virus infection. Further, AV2 (dimer) interaction with Cat2 might downregulated its expression and activity to prevent ROS dismutation, ultimately resulting in systemic necrosis. AV2 also suppress siRNA signals against PDS targeted by the host RNA silencing machinery. Green and red lines represent up- and downregulated transcripts, respectively.

Based on these observations, we propose that AV2 could act as multifunctional protein during ToLCPalV infection which is explained through a model (Fig. 8). AV2 predominantly exists as dimers and acts to suppress the host’s RNA silencing machinery. AV2 overexpression results in downregulation of the *Cat2* gene, which might be due to interaction between the AV2 and host Cat2. Downregulation of the *Cat2* gene might lead to higher accumulation of ROS and *PAD4* transcripts, which further primes SA production. SA creates a reducing environment that favours conversion of oligomeric NPR1 into monomers and promote their transport to the nucleus. Expression of NPR1 activates accumulation of *PR1* and *PR5* transcripts. AV2 expression in the host results in activation of SA signalling pathways; however, it appears that downregulation of the *EDS1* gene might result in disease susceptibility during viral infection. In conclusion, our study demonstrates that AV2 targets a host protein to establish the viral infection. Further investigations are required to understand the alterations of cell environment which favour virus replication, multiplication and spread.

## Materials and Methods

### Plant materials and virus strain

*N. benthamiana* and *S. lycopersicum cv*. Pusa Ruby were grown in an insect-free growth chamber at 24 °C and 70% relative humidity under 16 h light and 8 h darkness. The ToLCPalV strain used in the present study (DNA-A Accession no. AM884015 and DNA-B Accession no. AM992534) has been described previously^13^. Infectious clones of ToLCPalV (DNA-A and DNA-B) were previously constructed^23^. For the ToLCPalV∆AV2 construct, primers were designed to amplify DNA-A lacking 153 nt (51 aa) of the *AV2* gene without affecting the reading frame of the *CP* gene (Supplementary Fig. 6a). A 1.4-mer tandem repeat of DNA-A∆AV2 was cloned into the pCAMBIA1300 vector (Supplementary Fig. 6b). All recombinant plasmids (DNA-A, DNA-B and DNA-A∆AV2) were transformed into agrobacterium strain LBA4404^61^. Positive agrobacterium colonies were grown and resuspended in 2-morpholinoethanesulfonic acid (MES) buffer (pH 5.6); culture at OD_600_ = 0.8 was infiltrated into *N. benthamiana* and *S. lycopersicum* leaves using a needleless syringe. All experiments were performed twice in triplicates.

### Cloning of full-length and mutant *AV2* genes into the PVX vector

*AV2* full-length (366 bp) and N-terminal-deleted (336 bp) mutant genes were amplified and cloned in pGR106 (PVX vector; http://www.plantsci.cam.ac.uk/) at *Not*I and *Sal*I sites to generate PVX-AV2 and PVX-AV2∆10N constructs, respectively. *Agrobacterium* strain GV3101 was transformed with the PVX, PVX-AV2 and PVX-AV2∆10N plasmids and infiltrated into *N. benthamiana* at OD_600_ = 0.5.

### Stable expression of ToLCPalV AV2 in *N. benthamiana*

*AV2* was cloned under control of the CaMV35S promoter in the pCAMBIA1302 vector and transformed into agrobacterium strain LBA4404. *N. benthamiana* was transformed with agrobacterium culture using the leaf disc method^62^. Empty pCAMBIA1302 vector-transformed plants were used as the vector control. Regenerated transgenic plants were shifted in the soil, and phenotypes were observed under greenhouse conditions.

### Overexpression of AV2 in bacteria and antiserum production

To raise an AV2 antiserum, the viral gene was cloned in the pHIS parallel (provided by Dr. Peter Sheffield, University of Virginia (UVA)) expression vector at the *EcoR*I and *Xho*I restriction sites. AV2 protein expression was induced with 0.3 mM isopropyl β-D-1-thiogalactopyranoside (IPTG) for 4 h at 28 °C. Proteins were purified using Ni/NTA affinity chromatography and eluted with 250 mM imidazole buffer. For antiserum production, 500 mg purified AV2 protein and an equal volume of complete Freund’s adjuvant was injected into white New Zealander male rabbits for three consecutive weeks. For the booster dose, 1 mg of purified protein was emulsified with Freund’s incomplete adjuvant, and after 6 weeks, blood was taken from the marginal ear vein. Serum was collected, and IgG was purified using an IgG extraction kit (Bangalore, Genei, India).

### Southern blot and western blot analysis

Total DNA from *N. benthamiana* was isolated using CTAB reagent and treated with RNAase (Thermo scientific, USA). DNA was purified by Phenol: Chloroform: Isoamyl (PCI) extraction. For southern blotting, ten micrograms of total plant DNA was loaded on to 0.8% 1xTAE agarose gel and transferred to nitrocellulose membrane^63^. For detection of viral DNA, coat protein gene was used as probe and labeled with DIG-High Prime DNA labeling kit (Roche diagnostics, Germany). Membrane was hybridized with probe and detection was performed by CDP-star (Roche diagnostics, Germany). Protein extraction was done by pulverizing the leaves in liquid nitrogen and homogenized to fine powder. To this, 200 µl protein extraction solution (40 mM tris (hydroxymethyl)aminomethane hydrochloride (Tris-HCl) pH-7.4, 300 mM sodium chloride (NaCl), 5 mM magnesium chloride (MgCl_2_), 2 mM ethylenediaminetetraacetic acid (EDTA), 4 mM dithiothreitol (DTT), 0.5% Triton X-100, 1 mM phenylmethylsulfonyl fluoride (PMSF), 5% Glycerol and protease inhibitor cocktail) was added, samples were boiled for 5 minutes and subsequently loaded on 10% sodium dodecyl sulfate-polyacrylamide gel electrophoresis (SDS-PAGE). Electrophoresed protein samples were transferred to polyvinylidene difluoride (PVDF) membrane (Merck Millipore, USA) and detection was done by AV2 antiserum.

### Detection of ROS species

Hydrogen peroxide (H_2_O_2_) detection was done in *N. benthamiana* expressing AV2 protein and *S. lycopersicum* infected with ToLCPalV. Systemic leaves were immersed in DAB-HCl solution (pH-3.8) for 12 hrs. Treated leaves were bleached with acetic acid: glycerol: ethanol (1:1:3) in boiling water for 15 minutes and photographed^64^. H_2_O_2_ oxidizes the DAB in the presence of peroxidases to generate dark brown precipitate. Alternatively, presence of superoxide anion (O_2_^−^) was analyzed by Nitroblue tetrazolium (NBT) staining. NBT reacts with O2- to form a blue coloured formazan compound. Leaves were placed in 0.1% NBT dissolved in 10 mM Potassium phosphate buffer (pH-7.8) and 10 mM NaN_3_ and incubated in dark for 1 hour^65^. Stained leaf samples were decolorized in acetic acid: glycerol: ethanol (1:1:3) solution at 95 °C for 5 minutes and photographed.

### Yeast two-hybrid assay

For Y2H assays, full-length and N-terminal mutated *AV2* gene fragments were amplified using primer pairs carrying *EcoR*I and *Xho*I sites (Supplementary Table 1). Amplicons were cloned in the pGEMT®-T easy vector (Promega, Madison, US). Recombinant plasmids were further digested with *EcoR*I/*Xho*I, and the fragments were ligated into the pEG202 vector to generate LexA-AV2 fusion constructs. A tomato cDNA library was cloned into the pJG4-5 vector and fused with the B42 acid blob activation domain (B42) obtained from Dr. Yedidya Gafni, Volcani, centre Bet Dagan, Israel. Yeast transformation was carried out using the lithium acetate/Polyethylene glycol (LiOAc/PEG) method. Both LexA- and B42- fusion constructs were co-transformed in yeast strain EGY48, and colonies were selected on yeast nitrogen base galactose (Clontech, TakaraBio, Japan) plates lacking uracil, histidine, tryptophan and leucine (YNB (gal) –ura –trp –his –leu). Interaction selection was achieved by patching yeast colonies onto YNB (gal) –ura –trp –his –leu plates. Positive interactions were further confirmed by β-galactosidase activity by β-X-gal overlay assay. Since, LexA-AC4 and B42-SlOEE interacts in yeast (unpublished data); used as positive control while pHRFM1 (bicoid homeodomain) used as negative control in this study. On the basis of the Y2H data, full-length interactors were amplified from the tomato cDNA library using Phusion high-fidelity DNA polymerase (NEB labs, USA) and subsequently cloned into the pJET2.1 vector (Thermo Scientific, USA). Full-length interactor was further cloned into pJG4-5 to confirm the interaction observed in the Y2H assay.

### *In vivo* pull-down and the BiFC assay

To confirm Y2H interactions, genes encoding interactor proteins were cloned in the BiFC vectors pSPYCE(M), pSPYCE(MR), pSPYNE(R)173 and pSPYNE173 (a kind gift from Prof Jorg Kudla, Germany). The *AV2* and *SlCat2* genes were amplified using the *Spe*I/*Xho*I primer pair (Supplementary Table 1). *AV2* was cloned into pSPYCE(M) and pSPYCE(MR) vectors to produce AV2-cYFP and AV2-nYFP fusion, respectively. Similarly, *SlCat2* was cloned into pSPYNE(R)173 and pSPYNE173 to produce Cat2-nYFP and Cat2-cYFP fusion, respectively^66^. All BiFC constructs along with P19 suppressor (pBIN-P19) were introduced into *N. benthamiana* using agrobacterium strain GV3101. After 72 hours, emission of YFP fluorescence was detected at 514 nm excitation and 530–600 nm emission by confocal microscopy (Zeiss, Germany).

For *in vivo* pull-down assays, total plant protein was isolated from infiltrated leaves (AV2-HA+SlCat2-cMyc, SlCat2-cMyc+AC4-HA andAV2-HA-SlOEE-cMyc). Leaf samples were homogenized in protein extraction buffer (50 mM N-2-hydroxyethylpiperazine-N-2-ethane sulfonic acid-KOH (HEPES), 5 mM (EDTA), 1 mM (DTT), 10 mM sodium orthovanadate (Na_3_VO_4_), 5% glycerol and protease inhibitor). Protein samples were clarified by centrifugation, and 200 µg of protein extract was incubated with 2 µg of an anti-HA antibody (Clontech, Takara Bio, Japan) for 6 hours. Protein immunocomplexes were allowed to bind to sure bead protein G magnetic beads (Bio-Rad, USA) for 10 hours on a rocker at 4 °C. Elution of the immunoprecipitation products was completed in 1X Laemmli buffer, followed by incubation at 70 °C for 10 minutes. The eluted protein complexes were separated by 10% (SDS-PAGE), transferred to a PVDF membrane and blotted with an anti-cMYC antibody (Clontech, Takara Bio, Japan).

### Protein structure and interaction predictions

The amino acid sequences of Cat2 (LN880542) and AV2 (AM884015) were used to determine the protein structure through an ab initio modelling procedure using the I-TASSER server^67^ (http://zhang.bioinformatics.ku.edu/I-TASSER/). I-TASSER structural modelling is based on multiple threading alignments and iterations. Native structures are predicted on the basis of the highest confidence score (c-score). Structural similarity was measured by template modelling (TM-score) and the root-mean-square deviation (RMSD).

Docking studies were performed using a Hex protein docking server (Hex 8.0.0) with default values^68^ (http://www.loria.fr/ritchied/hex/). For docking studies, a 3D model of Cat2 was used as the receptor, and AV2 was used as the ligand. Both these inputs were loaded into the Hex program, and docking was carried out with the default parameters. For better representation, the Hex output was altered in PyMOL^69^ (https://www.pymol.org/view), and the colour scheme of both receptor and ligand was changed. The amino acids involved in the interaction between these two proteins, mediated by hydrogen bonds and hydrophobic contacts, was determined by LigPlot+^70^. Potentially involved hydrogen bonds are indicated by solid blue lines, and salt bridges are indicated by solid red lines between the residues involved. Non-bonded contacts are denoted by dashed orange lines.

### Yeast two-hybrid assay for AV2 protein self-interaction

A Y2H assay based on a split ubiquitin system was used to assess the self-interaction of AV2 protein. *AV2* was cloned into bait (pDHB1) and prey (pPR3N) vectors at the *Sfi*I site (Dualsystems AG, Switzerland). pDHB1-AV2 and pPR3N-AV2, pNubG-Fe65 and pTSU2-APP (positive control) and pDHB-AV2 and pPR3N (auto activation control) were co-transformed into yeast strain NMY51. Colonies were grown on YNB (glucose) selective medium supplemented with 10 mM 3-AT and but lacking adenine, leucine, tryptophan and histidine (-ade –leu –trp -his).

### Total RNA extraction and real-time PCR analysis

Total RNA was isolated from infected *N. benthamiana* leaves at different time points using the iRIS method^71^. Genomic DNA in the samples was digested with DNAase I (Thermo Scientific, USA). The concentration and purity of the RNA was determined using a Nanodrop 2000 (Thermo Scientific, USA). For first-strand cDNA synthesis, 2 µg of RNA was mixed with oligo-dT primer (New England Biolabs, USA) and briefly incubated at 70 °C for 5 minutes and then chilled on ice. Next, 40 mM dNTPs (10 mM each of dATP, dGTP, dTTP & dCTP), 5X M-MLV reaction buffer and enzyme (100 units) were added (Affymetrix, Santa Clara, USA). The tubes were kept at 37 °C for 75 minutes, and the enzyme was deactivated at 80 °C for 5 minutes in a thermocycler (Bio-Rad, USA). The primers used in this study were designed using Primer express 3.0.1 software (Life Technologies, USA), as described in Supplementary Table 1. qRT-PCR was performed using the Mx3000P^TM^ Real Time PCR system (Agilent Technologies, Santa Clara, USA) with 1:10 diluted cDNA, 0.2 µM forward and reverse primers and 1X DyNAmo Flash SYBR Green (Thermo Scientific, USA) in a final volume of 30 µl. The following thermal profile was used: 95 °C for 10 min followed by 40 cycles of 95 °C for 30 s, 60 °C for 30 s and 72 °C for 30 s. The reactions were set up in triplicate, and EF1α was used as an internal control^72^. A melting curve was generated for verification of primer specificity. All qRT-PCR reactions were performed with three biological replicates and three technical replicates. Data were analysed by normalizing Ct values to the internal control (EF1α) using the 2^−ΔΔCt^ method^73^.

### Statistical analysis of the data

Up- or downregulation of genes was considered on the basis of a ≥2-fold or <0.5-fold change in expression. To plot figures, the mean values of relative expression with respect to uninoculated controls were used. The standard error of three biological replicates is represented by the error bars. Student’s t-test was performed to identify statistical significance for gene expression between samples for each pair (mock and infected), with significant *(P < 0.05), highly significant **(P < 0.01) and very highly significant ***(P < 0.001) differences.

### TRV based gene silencing in *N. benthamiana*

The TRV vector and silencing controls for the silencing of *Cat2* in *N. benthamiana* were procured from Dr. Dinesh Kumar lab, UC Davis, USA. For reversal of gene silencing, *AV2* was cloned into the pTRV2 vector. The *Phytoene desaturase* (*PDS*) gene of *N. benthamiana* was cloned into the pTRV2 vector and used as the silencing control. The *P19* gene (suppressor) was cloned into the TRV2 vector at *Bam*HI and *Xho*I sites. For suppression of VIGS, equal ratios of TRV2-AV2+TRV2-PDS+TRV1, TRV2-P19+TRV2-PDS+TRV1 (suppressor control) and TRV2-PDS+TRV1 (VIGS control), agrobacterium cultures were infiltrated into *N. benthamiana*. The plants were kept in an insect-free growth chamber maintained at 23 °C under a 16-h light/8-h dark photoperiod, 65% relative humidity and 100 μmol/m^2^/s light intensity. For silencing of Cat2, approximately 300-bp fragments of *Cat2* were chosen for silencing using the SGN VIGS tool^74^. An approximately 300-bp *Cat2* fragment was cloned into the TRV2 vector at the *Eco*RI and *Xho*I sites. TRV2-Cat2, TRV1, and TRV2-PDS were agroinfiltrated into *N. benthamiana* as described above. Plants were challenged with ToLCPalV infection at 10 dpi of the VIGS experiment. For relative quantification of ToLCPalV in the samples^75^, primers complementary to *AC1* and *AV1* gene regions were designed. Total plant DNA of the above samples was used as the template in real-time PCR and the relative amount of virus present in the samples was calculated using the 2^−∆∆ct^ method^73^. *EF1α* was used as reference gene in the present study because it shows high expression stability in *N. benthamiana* upon virus infection^72^.

### CAT enzyme activity

Total Cat activity in wild and mutant ToLCPalV infected leaves was determined by dismutation of H_2_O_2_ by Cat enzyme at 240 nm^76^.

### Ethics Statement

All experiments for raising antisera were carried out in agreement with the guidelines approved by CSIR-IHBT. Experiments were approved by the Institutional Animal Ethics Committee under Committee for the Purpose of Control and Supervision on Experiment on Animals (CPCSEA).

### Ethical Approval And Informed Consent

For raising antisera, rabbits were used after approval from the Institutional Animal Ethics Committee under Committee for the purpose of control and supervision of experiments on animals (CPCSEA).

## Electronic supplementary material

Dataset 1

## References

[1] Dangl JL, Jones JD (2001). Plant pathogens and integrated defence responses to infection. Nature.

[2] Lamb C, Dixon RA (1997). The oxidative burst in plant disease resistance. Annu. Rev. Plant Physiol. Plant Mol. Biol..

[3] Mandadi KK, Scholthof KG (2013). Plant immune response against viruses: How does a virus cause disease?. Plant Cell.

[4] Vranova E, Inze D, Van Breusegem F (2002). Signal transduction during oxidative stress. J. Exp. Bot..

[5] Willekens H, Inze D, Van Montagu M, Van Camp W (1995). Catalases in plants. Mol. Breed..

[6] Ding SW, Voinnet O (2007). Antiviral immunity directed by small RNAs. Cell.

[7] Vanitharani R, Chellappan P, Pita J, Fauquet CM (2004). differential roles of AC2 and AC4 of cassava geminiviruses in mediating synergism and suppression of post transcriptional gene silencing. J. Virol..

[8] Trinks D (2005). Suppression of RNA silencing by a geminivirus nuclear protein, AC2, correlates with transactivation of host genes. J. Virol..

[9] Glick E (2008). Interaction with host SGS3 is required for suppression of RNA silencing by *Tomato yellow leaf curl virus* V2 protein. Proc. Natl. Acad. Sci. USA.

[10] Zhang J, Dong J, Xu Y, Wu J (2012). V2 protein encoded by *Tomato leaf curl China virus* is an RNA silencing suppressor. Virus Res..

[11] Zerbini FM (2017). ICTV Virus Taxonomy Profile: Geminiviridae. J. of Gen. Virol..

[12] Fauquet CM (2003). Revision of taxonomic criteria for species demarcation in the family Geminiviridae, and an updated list of begomovirus species. Arch. Virol..

[13] Kumar Y, Hallan V, Zaidi AA (2008). Molecular characterization of a distinct bipartite begomovirus species infecting tomato in India. Virus Genes.

[14] Malik AH, Briddon RW, Mansoor S (2011). Infectious clones of *Tomato leaf curl Palampur* virus with a defective DNA B and their pseudo recombination with *Tomato leaf curl New Delhi virus*. Virol. J..

[15] Heydarnejad J, Hesari M, Massumi H, Varsani A (2013). Incidence and natural hosts of Tomato leaf curl Palampur virus in Iran. Aus. Plant Pathol..

[16] Rojas MR (2001). Functional Analysis of Proteins Involved in Movement of the Monopartite Begomovirus, Tomato Yellow Leaf Curl Virus. Virology.

[17] Zrachya A (2007). Suppressor of RNA silencing encoded by Tomato yellow curl virus-Israel. Virology.

[18] Sharma P, Ikegami M (2010). Tomato leaf curl java virus V2 protein is a determinant of virulence, hypersensitive response and post transcriptional gene silencing. Virology.

[19] Luna AP, Morilla G, Voinnet O, Bejarano ER (2012). Functional analysis of gene-silencing suppressors from tomato yellow leaf curl disease viruses. Mol. Plant. Microbe Interact..

[20] Fukunaga R, Doudna JA (2009). dsRNA with 5′ overhangs contributes to endogenous and antiviral RNA silencing pathways in plants. EMBO J..

[21] Bar-Ziv A (2012). The tomato yellow leaf curl virus (TYLCV) V2 protein interacts with the host papain-like cysteine protease CYP1. Plant Signal Behav..

[22] Moshe A (2015). The Tomato yellow leaf curl virus V2 protein forms aggregates depending on the cytoskeleton integrity and binds viral genomic DNA. Sci. Rep..

[23] Kumar, Y. Genome Organization and Mechanism of RNA Silencing Suppression of Begomoviruses Infecting Some Solanaceous Crops. http://hdl.handle.net/10603/10222 (2011).

[24] Regelsberger GC (2001). The role of distal tryptophan in the bifunctional activity of catalase-peroxidases. Biochem. Soc. Trans..

[25] Mittler R (2002). Oxidative stress, antioxidants and stress tolerance. Trends Plant Sci..

[26] Mhamdi A (2010). Catalase function in plants: a focus on Arabidopsis mutants as stress-mimic models. J. Exp. Bot..

[27] Varma A, Malathi VG (2003). Emerging geminivirus problems: A serious threat to crop production. Ann. Appl.Biol..

[28] Matic S, Pegoraro M, Noris E (2016). The C2 protein of tomato yellow leaf curl Sardinia virus acts as a pathogenicity determinant and a 16-amino acid domain is responsible for inducing a hypersensitive response in plants. Virus Res..

[29] Hussain M, Mansoor S, Iram S, Fatima AN, Zafar Y (2005). The Nuclear Shuttle Protein of Tomato Leaf Curl New Delhi Virus Is a Pathogenicity Determinant. J. of Virol..

[30] Kang SH, Qu F, Morris TJ (2016). A spectrum of HRT-dependent hypersensitive responses elicited by the 52 amino acid N-terminus of turnip crinkle virus capsid protein and its mutants. Virus Res..

[31] Sampere RN (2016). Pepino mosaic virus RNA-Dependent RNA Polymerase POL Domain Is a Hypersensitive Response-Like Elicitor Shared by Necrotic and Mild Isolates. Phytopathology.

[32] Carr JP, Lewsey MG, Palukaitis P (2010). Signaling in induced resistance. Adv. Virus Res..

[33] Chu M, Desvoyes B, Turina M, Noad R, Scholthof HB (2000). Genetic dissection of Tomato bushy stunt virus p19-protein-mediated host-dependent symptom induction and systemic invasion. Virology.

[34] Komatsu K (2010). Viral-Induced Systemic Necrosis in Plants Involves Both Programmed Cell Death and the Inhibition of Viral Multiplication, Which Are Regulated by Independent Pathways. MPMI.

[35] Inaba J, Kim BM, Shimura H, Masuta C (2011). Virus induced necrosis is a consequence of direct protein- protein interaction between a viral RNA silencing suppressor and a host catalase. Plant Physiol..

[36] Murota K, Shimura H, Takeshite M, Masuta C (2016). Interaction between Cucumber mosaic virus 2b protein and plant catalase induces a specific necrosis in association with proteasome activity. Plant Cell Rep.

[37] Mathioudakis MM (2013). Pepino mosaic virus triple gene block protein 1 (TGBp1) interacts with and increases tomato catalase 1 activity to enhance virus accumulation. Mol. Plant Pathol..

[38] Hanley-Bowdin L, Bejarano ER, Robertson D, Mansoor S (2013). Geminiviruses: masters at redirecting and reprogramming plant processes. Nat. Rev. Microbiol..

[39] Padidam M, Beachy RN, Fauquet CM (1996). The role of AV2 (‘precoat’) and coat protein in viral replication and movement in tomato leaf curl geminivirus. Virology.

[40] Hak H (2015). TYLCV-Is movement in planta does not require V2 protein. Virology.

[41] Wartig L, Kheyr-Pur A, Noris E, De Kouchkovsky F, Jouanneau F (1997). Genetic analysis of the monopartite tomato yellow leaf curl geminivirus: roles of V1, V2 and C2 ORFs in viral pathogenesis. Virology.

[42] Ramirez ER, Sudarshana MR, Lucas WJ, Gilbertson RL (2000). Bean dwarf mosaic virus BV1 Protein Is a Determinant of the Hypersensitive Response and Avirulence in Phaseolus vulgaris. Mol. Plant Microbe Int..

[43] Cui X, Tao X, Xie Y, Fauquet CM, Zhou XA (2004). DNAβ Associated with Tomato Yellow Leaf Curl China Virus Is Required for Symptom Induction. J. of Virol..

[44] Van Loon LC, Rep M, Pietersen CMJ (2006). Significance of inducible defense-related proteins in infected plants. Annu. Rev. Phytopathol..

[45] Penninckx IA (1996). Pathogen-induced systemic activation of a plant defensing gene in Arabidopsis follows a salicylic acid-independent pathway. The Plant Cell.

[46] Thiel H, Hleibieh K, Gilmer D, Varrelmann M (2012). The P25 Pathogenicity Factor of Beet necrotic yellow vein virus Targets the Sugar Beet 26S Proteasome Involved in the Induction of a Hypersensitive Resistance Response via Interaction with an F-box Protein. MPMI.

[47] Kim BM, Suehiro N, Natsuaki T, Inukai T, Masuta C (2010). The P3 Protein of Turnip mosaic virus Can Alone Induce Hypersensitive Response-Like Cell Death in Arabidopsis thaliana Carrying TuNI. MPMI.

[48] Mou Z, Fan WH, Dong XN (2003). Inducers of plant systemic acquired resistance regulate NPR1 function through redox changes. Cell.

[49] Wang D, Weaver ND, Kesarwani M, Dong X (2005). Induction of protein secretory pathway is required for systemic acquired resistance. Science.

[50] Garcion C (2008). Characterization and biological function of the ISOCHORISMATE SYNTHASE2 gene of Arabidopsis. Plant Physiol..

[51] Wiermer M, Feys BJ, Parker JE (2005). Plant immunity: the EDS1 regulatory node. Curr. Opin. Plant Biol..

[52] Breitenbach HH (2014). Contrasting Roles of the Apoplastic Aspartyl Protease Apoplastic, Enhanced Disease Susceptibility1-Dependent1 and Legume Lectin-Like Protein1 in Arabidopsis Systemic acquired resistance. Plant Physiol..

[53] Zhou T (2014). Domains of the cucumber mosaic virus 2b silencing suppressor protein affecting inhibition of salicylic acid-induced resistance and priming of salicylic acid accumulation during infection. J. Gen. Virol..

[54] Roshan, P., Kulshreshtha, A. & Hallan, V. Identification of host cellular targets of AC4 and AV2 proteins of tomato leaf curl palampur virus and their sub-cellular localization studies. *Virus Dis*. 10.1007/s13337-017-0405-5. 10.1007/s13337-017-0405-5 PMC5747847 29291230

[55] Sueda K (2010). The C-terminal residues of the 2b protein of Cucumber mosaic virus are important for efficient expression in Escherichia coli and DNA-binding. FEBS Lett..

[56] Cui X, Li G, Wang D, Hu D, Zhou X (2005). A Begomovirus DNAβ-Encoded Protein binds DNA, Functions as a Suppressor of RNA Silencing, and Targets the Cell Nucleus. J. Virol..

[57] Yang J (2008). βC1, the pathogenicity factor of TYLCCNV, interacts with AS1 to alter leaf development and suppress selective jasmonic acid responses. Genes Dev..

[58] Bhattacharyya, D. *et al*. A geminivirus betasatellite damages the structural and functional integrity of chloroplasts leading to symptom formation and inhibition of photosynthesis. *J. Exp. Bot*. 10.1093/jxb/erv299 (2015). 10.1093/jxb/erv299 PMC4566980 26113193

[59] Chamnongpol S (1996). Transgenic tobacco with a reduced catalase activity develops necrotic lesions and induces pathogenesis-related expression under high light. The Plant J..

[60] Huang Z (2005). Salicylic Acid-Dependent Expression of Host Genes in Compatible Arabidopsis-Virus Interactions. Plant physiol..

[61] Weigel D, Glazebrook J (2006). Transformation of agrobacterium using the freeze-thaw method. Cold Spring Harbor Protocols.

[62] Clemente, T. Nicotiana (Nicotiana tobaccum, Nicotiana benthamiana) ed. Wang K. 143–154 (Humana press, New Jersey, 2006). 10.1385/1-59745-130-4:143 16988341

[63] Sambrook, J., Fritsch, E. & Maniatis, T. Molecular cloning: a Laboratory Manual. New York. USA: Cold Spring Harbour Press (1989).

[64] Daudi A, Brien JA (2012). Detection of Hydrogen Peroxide by DAB staining in Arabidopsis leaves. Bio-protocol.

[65] Kawai-Yamada M, Ohori Y, Uchimiya H (2004). Dissection of Arabidopsis Bax inhibitor-1 suppressing Bax-, hydrogen peroxide-, and salicylic acid-induced cell death. Plant Cell.

[66] Waadt R (2008). Multicolor bimolecular fluorescence complementation reveals simultaneous formation of alternative CBL/CIPK complexes in planta. Plant J..

[67] Zhang Y (2008). I-TASSER server for protein 3D structure prediction. BMC Bioinform..

[68] Macindoe G, Mavridis L, Venkatraman V, Devignes MD, Ritchie DW (2010). HexServer: an FFT-based protein docking server powered by graphics processors. Nucleic Acid Res..

[69] The PyMOL Molecular Graphics System, Version 1.8 Schrödinger, LLC.

[70] Laskowski RA, Swindells MB (2011). LigPlot+: multiple ligand-protein interaction diagrams for drug discovery. J. Chem. Inf. Model..

[71] Ghawana S (2011). An RNA isolation system for plant tissues rich in secondary metabolites. BMC Res. Notes.

[72] Liu D (2012). Validation of Reference Genes for Gene Expression Studies in Virus-Infected Nicotiana benthamiana Using Quantitative Real-Time PCR. PLoS ONE.

[73] Livak K, Schmittgen T (2001). Analysis of relative gene expression data using real-time quantitative PCR and the 2^−ΔΔCt^ method. Methods..

[74] Pozo N, Rosli HG, Martin GB, Mueller LA (2015). The SGN VIGS Tool: User-Friendly Software to Design Virus-Induced Gene Silencing (VIGS) Constructs for Functional Genomics. Mol. Plant.

[75] Noris E, Miozzi L (2015). Real-time PCR protocols for the quantification of the begomovirus tomato yellow leaf curl Sardinia virus in tomato plants and in its insect vector. Methods Mol. Biol..

[76] Aebi, H. Catalase *in Vitro. Methods in Enzymology*. Colowick SP, Kaplan NO, editors. Vol. 105. Florida: Acad. Press pp. 114–121 10.1016/s0076-6879(84)05016-3 6727660

