# Supplementary material for: AV2 protein of tomato leaf curl Palampur virus promotes systemic necrosis in Nicotiana benthamiana and interacts with host Catalase2
Source: Sci Rep. 2018 Jan 19;8:1273. doi: 10.1038/s41598-018-19292-3 (PMC5775426; doi:10.1038/s41598-018-19292-3)
Supplement: Supplementary file 1 — Dataset 1 [file 41598_2018_19292_MOESM1_ESM.doc]

**Supplementary Information**

**AV2 protein of *Tomato leaf curl Palampur virus* promotes systemic necrosis in *Nicotiana benthamiana* and interacts with host catalase2**

Poonam Roshan ^1,2^, Aditya Kulshreshtha^1,2^, Surender Kumar^1,2^, Rituraj Purohit^1,3^ and Vipin Hallan^1,2^*

1- Academy of Scientific & Innovative Research (AcSIR), CSIR-Institute of Himalayan Bioresource Technology, Palampur, HP, 176061, India

2- Plant Virology Lab, CSIR-IHBT, Palampur, HP, 176061, India

3- Biotechnology division, CSIR-IHBT, Palampur, HP, 176061, India

Corresponding author: Vipin Hallan, Plant Virology Lab, CSIR-Institute of Himalayan Bioresource Technology, Palampur, HP, 176061, India

E-mail: hallan@ihbt.res.in; [rnaivi@gmail.com](mailto:rnaivi@gmail.com)

Telephone: +91-1894-233339


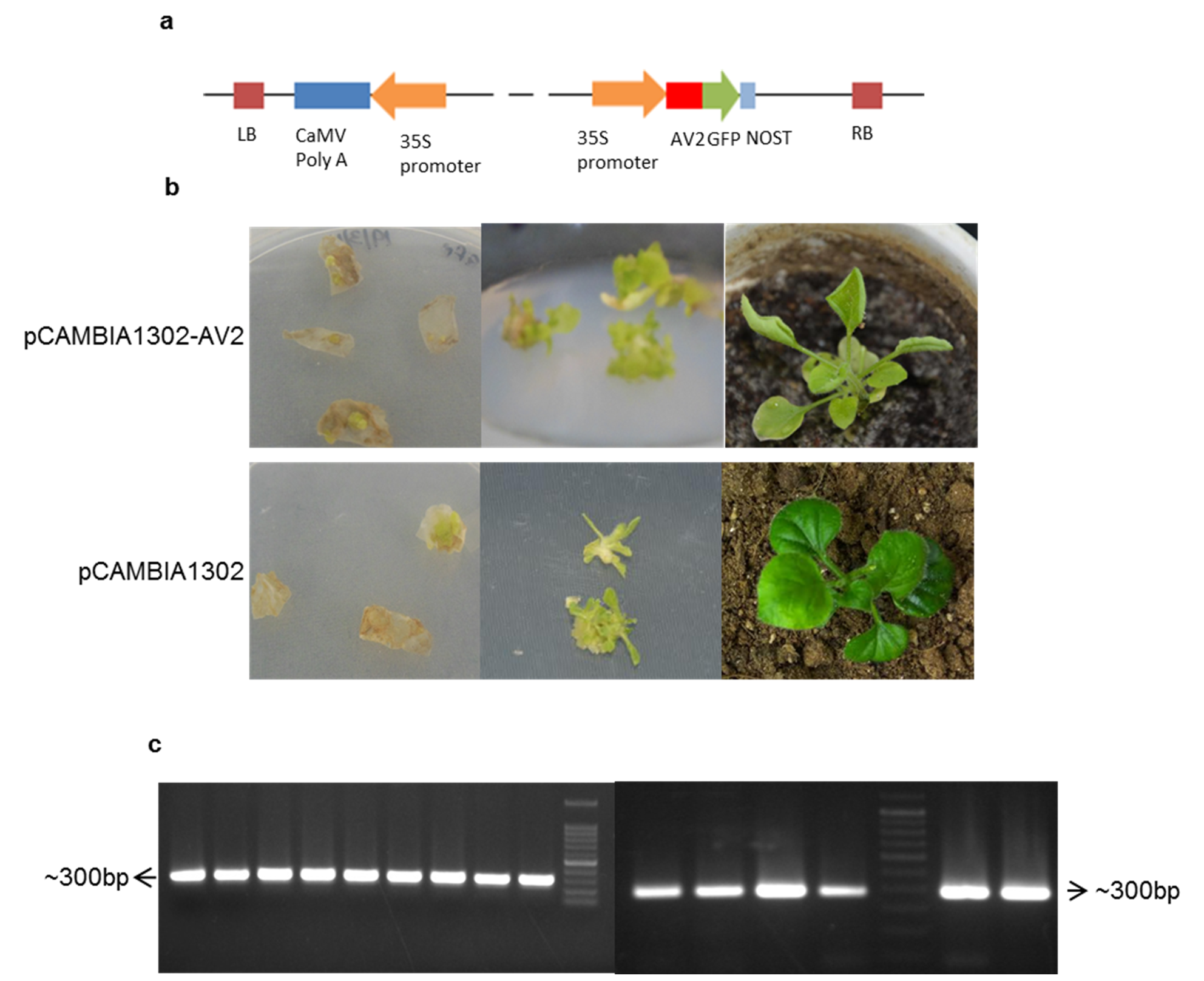


**Supplementary Figure S1:** (a) Diagrammatic representation of *AV2* gene in pCAMBIA1302 vector. (b) Regeneration of callus into T0 lines after leaf disc transformation of pCAMBIA-1302-AV2 and pCAMBIA1302 (vector control). (c) PCR amplification for the presence of AV2 transgene in T0 lines using *AV2* specific primers.


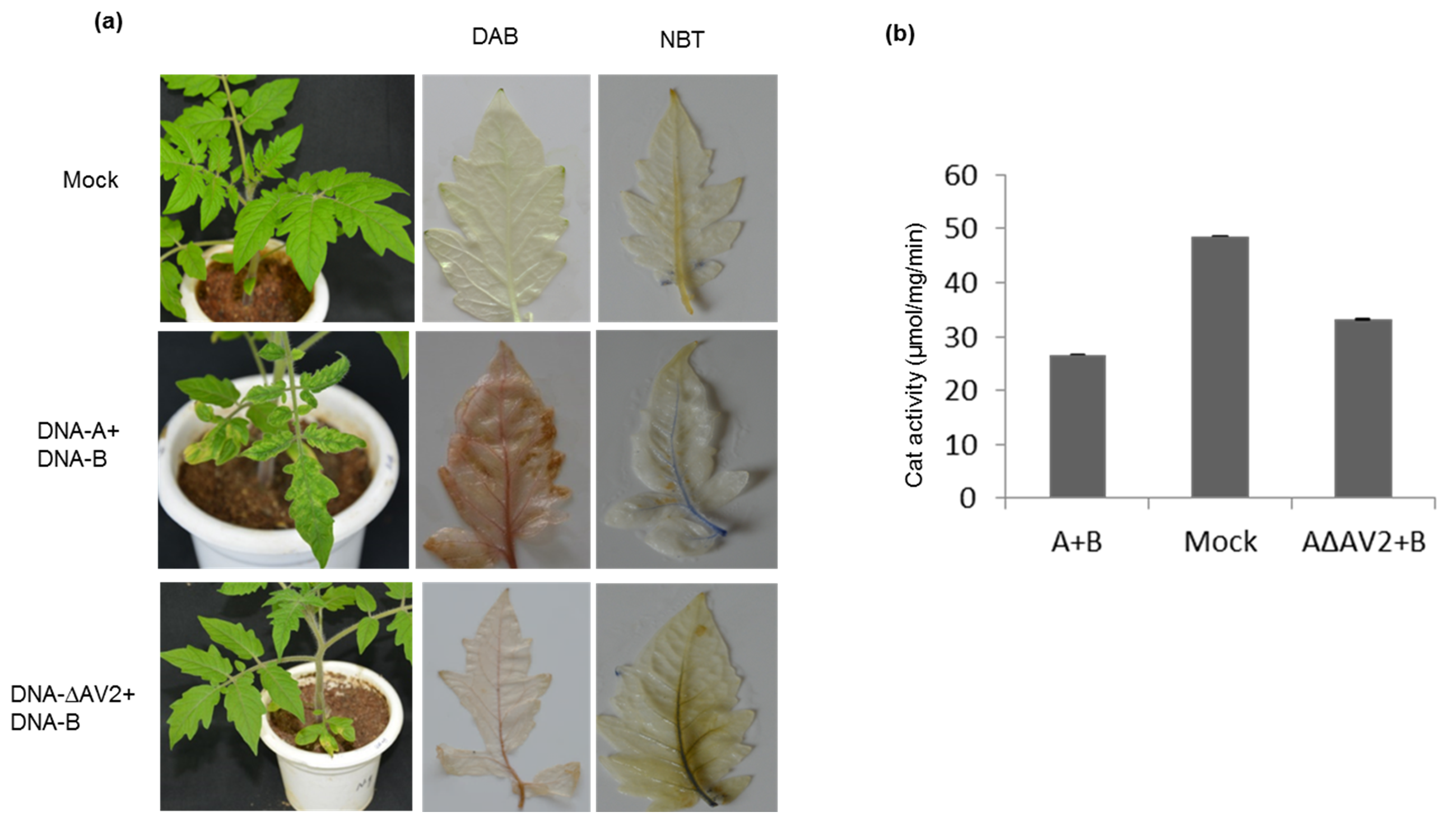


**Supplementary Figure S2:** (a) DAB and NBT staining of *S. lycopersicum* inoculated with DNA-A+DNA-B and DNA-A∆AV2+DNA-B at 21dpi; showing higher accumulation of ROS in DNA-A+ DNA-B infiltrated samples. (b) Total Catalase activity in systemic leaves of *N. benthamiana* infiltrated with A+B, A∆AV2+B and mock at 21 dpi.


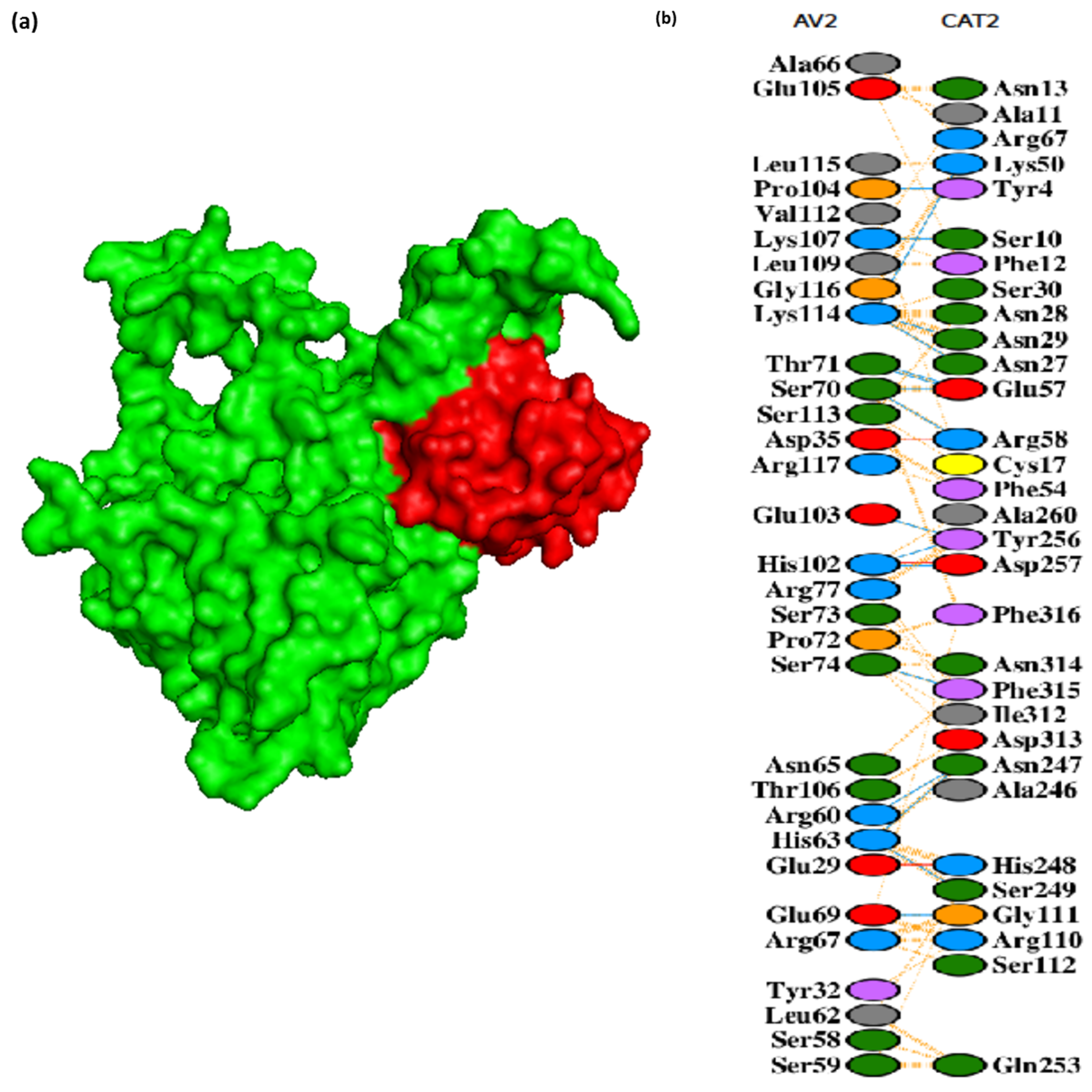


**Supplementary Figure S3:** Residual interactions at the protein–protein interface in the SlCat2-AV2 complex. The coding scheme is as follows: H-bonds are represented in blue colour; non-bonded interactions are represented with dashed orange lines. Positively charged residues are represented in blue colour; negatively charged residues are in red colour; neutral residues are represented in green. Aliphatic and aromatic residues are represented in grey and purple, respectively. Proline and glycine are represented in brown and cysteine in yellow.

**Supplementary Figure S4:** Yeast transformants were streaked on YNB (glu) –ade –leu –trp –his medium supplemented with 10 mM 3-AT showing the self-association of AV2. (a) auto-activation control (pDHB-AV2+pPR3N); (b) pNubG-Fe65+pTSU2-APP (positive control); (c) pDHB1-AV2+ pPR3N-AV2


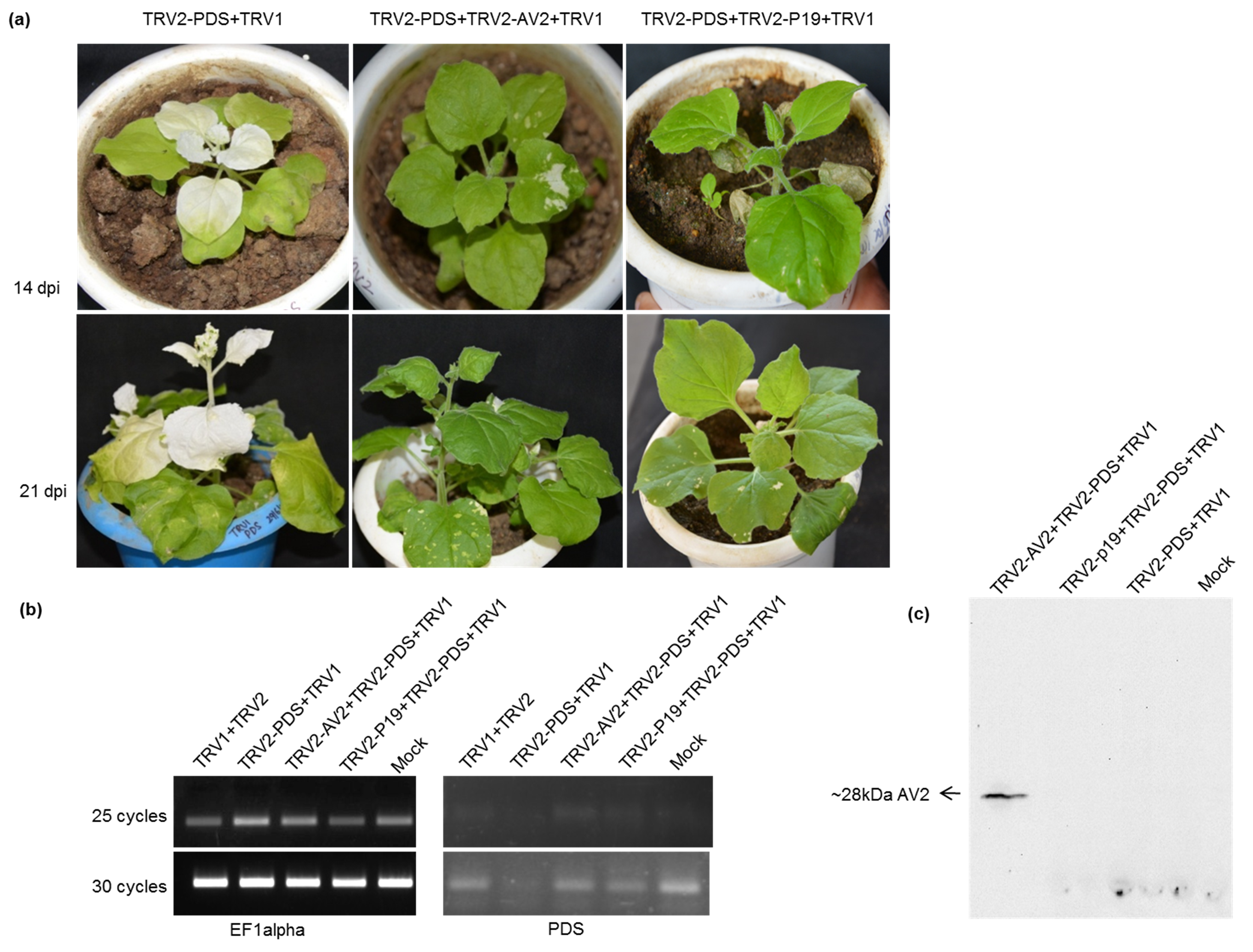


**Supplementary Figure S5:** (a) Bleaching phenotype in *N. benthamiana* leaves due to silencing of PDS gene (left); reduction of bleaching phenotype was observed when TRV2-AV2 co-inoculated with TRV2-PDS (middle) and TRV2-P19 co-inoculated with TRV2-PDS as suppressor control at 14 & 21 dpi, respectively (b) Semi quantitative PCR of the above samples to confirm suppression of VIGS (c) Western blot to detect presence of AV2; dimer form (~28kDa) was detected in systemic leaves of TRV2-PDS+ TRV2-AV2+TRV1 infiltrated plants.


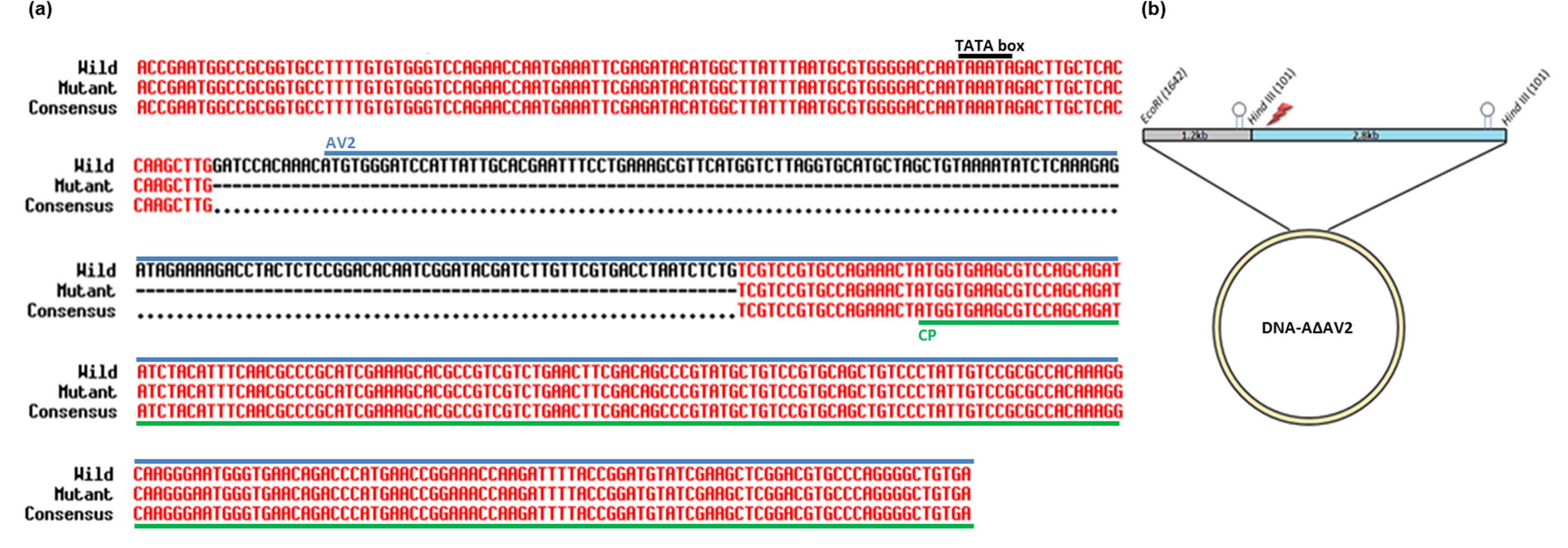


**Supplementary Figure S6:** (a) Sequence alignment of wild and mutant DNA-A depicting the 153 nt deletion in the AV2 gene; and (b) Agroinfectious clone strategy of DNA-A ∆AV2 infectious clone.

**Supplementary Table 1:** List of primers used in the present study

| **Name** | **Sequence (5’-3’)** | **Use** |
| --- | --- | --- |
| AV2 *Not*IFP | GCGGCCGCATGTGGGATCCATTATTG | PVX expression |
| AV2 Δ10*Not*IFP | GCGGCCGCATGGAAAGCGTTCATGG | PVX expression |
| AV2 *Sal*I RP | GTCGACCAGCCCCTGGGCACGTCC | PVX expression |
| AV2 Δ10 *Sal*I RP | GTCGACTCACAGCCCCTGGGCACGTCC | PVX expression |
| AV2 Δ20 *Sal*I RP | GTCGACCAGCCCCTGGGCACGTCC | PVX expression |
| AV2 *EcoR*I FP | CCCGAATTCATGTGGGATCCATTATTGCACGAATT | Y2H/TRV/pET28expression |
| AV2*Xho*I RP | CTTCTCGAGTCACAGCCCCTGGGCACGTC | Y2H/ pET28expression |
| AV2Δ10*EcoR*I FP | CTGGAATTCATGGAAAGCGTTCATGGTCTTAG | Y2H |
| AV2*Xho*I RP | CTCGAGCAGCCCCTGGGCACGTC | TRV expression/ BiFC |
| AV2 *Spe*I FP | ACTAGTATGTGGGATCCATTAT | BiFC |
| SlCAT2 *Spe*I FP | ACTAGTATGGATCCTTACAAGTACC | BiFC |
| SlCAT2 *Xho*I RP | CTCGAGTATGCTTGGTCTCAC | BiFC |
| SlCAT2 *EcoR*I FP | GAATTCATGGATCCTTACAAGTACC | Y2H |
| SlCAT2 *Xho*I RP | CTCGAGTCATATGCTTGGTCTCAC | Y2H |
| NbPR1 QFP | AATAGGGTAGCGGCCTTTGC | Q PCR |
| NbPR1 QRP | CGGCGGCTAGGTTTTCG | Q PCR |
| NbPR5 QFP | TGGATTTAACATTCCTATGTCTTTTGG | Q PCR |
| NbPR5 QRP | ACCTGGGCATTCACCATTTATATT | Q PCR |
| Nb NPR1 QFP | TGATGATGTTGAATTACTGCAGATGT | Q PCR |
| Nb NPR1 QRP | GCTGTAGTCTTTGCATCGCAAT | Q PCR |
| Sl PAL QFP | TGGCTTCTTACTGCTCGGAACT | Q PCR |
| Sl PAL QRP | ATTAAGCCCAAGGAATTCACATCT | Q PCR |
| Sl PAD4 QFP | CGATAAGTGGTGGAAAACTGAGAA | Q PCR |
| Sl PAD4 QRP | CTCAACGCGAGCCCAAAA | Q PCR |
| Sl EDS1 QFP | TTGGATCCCCTCTTGTTGGTAA | Q PCR |
| Sl EDS1 QRP | GACAACGTCGTACTTCATGACAAAA | Q PCR |
| Sl ICS1 QFP | CATTCGCCGGATTCATT | Q PCR |
| Sl ICS1 QRP | CCACCCCTAGCCCGTGTT | Q PCR |
| Sl PR1 QFP | TGCAAAATTCACCCCAAGACT | Q PCR |
| Sl PR1 QRP | GCTCGGGATGCCAAGTTG | Q PCR |
| Sl NPR1 QFP | TAGTATATGCTGCATGAACGAATCG | Q PCR |
| Sl NPR1 QRP | CGCAGACGCATCGAAGATAG | Q PCR |
| Sl PR5 QFP | CGTCTCGAGAGAGGTCAAAGTTG | Q PCR |
| Sl PR5 QRP | TACCAGCACCATCAAAATTGCA | Q PCR |
| Sl EF1α QFP | GATTGACAGACGTTCTGGTAAGGA | Q PCR |
| Sl EF1α QRP | ACCGGCATCACCATTCTTCA | Q PCR |
| Nb EF1α QFP | AGCTTTACCTCCCAAGTCATC | Q PCR |
| Nb EF1α QRP | AGAACGCCTGTCAATCTTGG | Q PCR |
| Nb Cat2 QFP | GGGTGGAGGCCTTGTCTGAT | Q PCR |
| Nb Cat2 QRP | AGCAAGCTTTTGACCCAGAGAT | Q PCR |
| AV2QFP | TGTCGTCCGTGCCAGAAAC | Q PCR |
| AV2QRP | TACGGGCTGTCGAAGTTCAGA | Q PCR |
| AC1QFP | TGAAGGAAAATTCCAGTGCAAA | Q PCR |
| AC1QRP | CGCTGATTTAGCTCCCTGAATG | Q PCR |
| AV1QFP | TCAAGACCAAGAACCACACGAAT | Q PCR |
| AV1QRP | AACATATTAAAAACTTCGCCGAAATC | Q PCR |
| Cat VIGS FP | GAATTCATCGACTACTTCCCTTC | VIGS Silencing |
| Cat VIGS RP | CTCGAGTCTCACATTAAGCCTAG | VIGS Silencing |
